# Temporal and spatial earthquake clustering revealed through comparison of millennial strain-rates from ^36^Cl cosmogenic exposure dating and decadal GPS strain-rate

**DOI:** 10.1038/s41598-021-02131-3

**Published:** 2021-12-02

**Authors:** Francesco Iezzi, Gerald Roberts, Joanna Faure Walker, Ioannis Papanikolaou, Athanassios Ganas, Georgios Deligiannakis, Joakim Beck, Soeren Wolfers, Delia Gheorghiu

**Affiliations:** 1 DiSPUTer, Università Degli Studi “Gabriele d’Annunzio” Chieti-Pescara, Via dei Vestini, 66100 Chieti, Italy; 2 Department of Earth and Planetary Sciences, Birkbeck, University of London, Malet Street, London, WC1E 7HX UK; 3 Institute for Risk and Disaster Reduction, University College London, Gower Street, London, WC1E 6BT UK; 4 Mineralogy-Geology Laboratory, Department of Natural Resources Development and Agricultural Engineering, Agricultural University of Athens, 75 Iera Odos, 118-55 Athens, Greece; 5 Institute of Geodynamics, National Observatory of Athens, Lofos Nymfon, 11810 Athens, Greece; 6 Computer, Electrical and Mathematical Sciences & Engineering (CEMSE), King Abdullah University of Science and Technology (KAUST), Thuwal, 23955-6900 Kingdom of Saudi Arabia; 7 Scottish Universities Environmental Research Centre, Rankine Avenue, East Kilbride, G750QF UK

**Keywords:** Natural hazards, Solid Earth sciences

## Abstract

To assess whether continental extension and seismic hazard are spatially-localized on single faults or spread over wide regions containing multiple active faults, we investigated temporal and spatial slip-rate variability over many millennia using in-situ ^36^Cl cosmogenic exposure dating for active normal faults near Athens, Greece. We study a ~ NNE-SSW transect, sub-parallel to the extensional strain direction, constrained by two permanent GPS stations located at each end of the transect and arranged normal to the fault strikes. We sampled 3 of the 7 seven normal faults that exist between the GPS sites for ^36^Cl analyses. Results from Bayesian inference of the measured ^36^Cl data implies that some faults slip relatively-rapidly for a few millennia accompanied by relative quiescence on faults across strike, defining out-of-phase fault activity. Assuming that the decadal strain-rate derived from GPS applies over many millennia, slip on a single fault can accommodate ~ 30–75% of the regional strain-rate for a few millennia. Our results imply that only a fraction of the total number of Holocene active faults slip over timescales of a few millennia, so continental deformation and seismic hazard are localized on specific faults and over a length-scale shorter than the spacing of the present GPS network over this time-scale. Thus, (1) the identification of clustered fault activity is vital for probabilistic seismic hazard assessments, and (2) a combination of dense geodetic observations and palaeoseismology is needed to identify the precise location and width of actively deforming zones over specific time periods.

## Introduction

Identification of the locations of active faults and associated slip-rates is key to constraining processes responsible for continental deformation^1^ and seismic hazard^2^. However, earthquake activity can be clustered in space and time^3^, so it is desirable to gain observations constraining the deformation over different time-scales and spatial-scales^4–6^. For example, it is common for regional strain-rates to be mapped using decadal observations from the Global Positioning System (GPS) and Global Navigation Satellite Systems (GNSS)^7–13^. However, debate is centered on whether such results (a) apply over multiple seismic cycles (hundreds to many thousands years), and (b) can resolve the locations of faults responsible for the observed strain given spatially-clustered activity and the available geodetic station spacing^14–17^. This leads to uncertainty in defining (1) the locations of active source faults for seismic hazard analysis and their deformation rates, and (2) the regions within which strain rates can be used to derive the rheology and hence dynamics of continental deformation, involving the links between body forces, tectonic forcing, and the dynamic viscosity of the deforming region and the fault/shear-zones it contains^18^.

Palaeoseismological observations provide key observations that are needed to constrain the above. Results have been interpreted to suggest that earthquake activity on a single fault can be clustered in time, with clusters containing several large-magnitude earthquakes (M_w_ > 6.0) and periods of quiescence lasting decades to several millennia^3,6,19–31^. These studies, some involving trenching and ^14^C dating of offset stratigraphy, and some involving ^36^Cl studies of fault planes, suggest that earthquake activity may swap across strike onto neighbouring faults through time^21,24,25,27^. This process would allow faults to share the regional strain-rate through time through non-synchronous pulses of high or low slip-rate on faults distributed parallel to the regional principal strain direction^32,33^. Although a number of studies compare millennial fault slip rates with decadal geodetic strain-rates^16,17,34^, examples where geodetic strain-rates are compared with strain-rates derived from slip-rate pulses lasting few millennia on individual faults arranged parallel to the regional principal strain orientation are scarce. Cosmogenic ^36^Cl dating offers the opportunity to accomplish the above because it provides long (Holocene and longer) continuous records of fault slip-rate, covering multiple seismic cycles. Thus, ^36^Cl measurements allow a test of whether geodetic strain-rates associated with normal faulting apply over longer timescales, defining the precise locations of all actively-slipping faults in precise time periods within wide zones of continental deformation.

**Figure 1:**
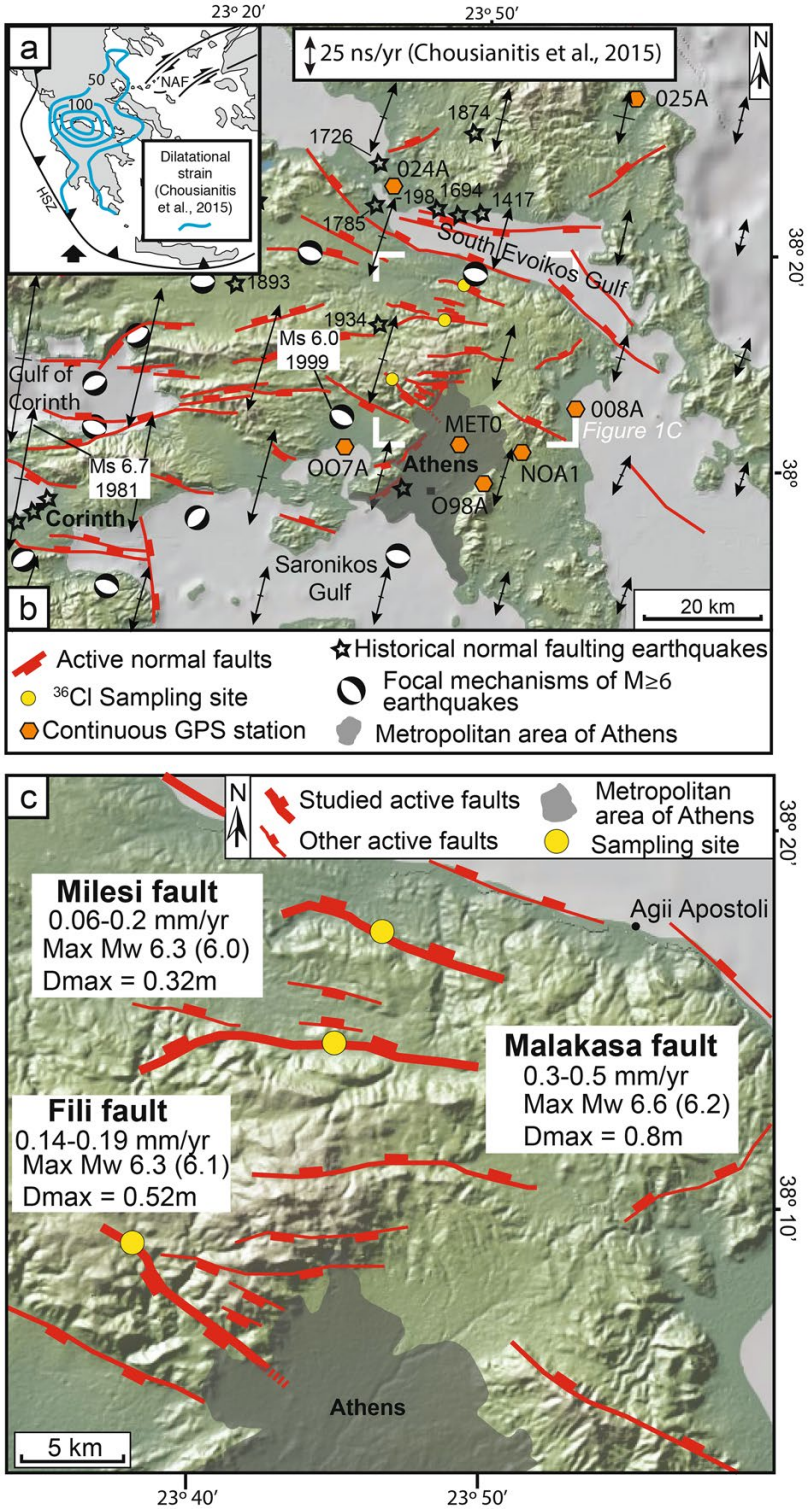
Location maps of the study area. (**a**) Regional setting of Greece with dilatational strains from GPS data (from Chousianitis et al.^10^). HSZ: Hellenic Subduction Zone; NAF: North Anatolian Fault. (**b**) Fault map with red lines indicating active faults, modified from Deligiannakis et al.^49^ and Konstantinou et al.^34^. Black lines with double arrowheads are principal horizontal strain-rate axes on a uniform grid inverted from the GPS velocity field, averaged over a grid used for calculations (see Chousianitis et al.^10^, for details). Focal mechanisms of earthquakes are from Chousianitis et al.^10^ and references therein. Historical earthquakes are from Grutzner et al.^46^, and reference therein. Yellow circles are locations of the 36Cl sampling sites. (**c**) Detailed fault map of Attica, with highlighted the sampled faults (attributes are from Deligiannakis et al.^49^, magnitudes in brackets are from Konstantinou et al.^34^; fault traces are modified from Deligiannakis et al.^49^ and Konstantinou et al.^34^). Thick red lines are the sampled faults; thin red lines are other active faults.

This paper presents ^36^Cl results from three sub-parallel principal active faults arranged orthogonal to the direction of the geodetically-derived principal extensional strain in Attica, central Greece (Fig. 1). The analysis of Holocene slip-rates, geodetic data and relocated instrumental seismicity shows that a number of normal faults in Attica are active^34^. However, existing data are insufficient to assess whether earthquake clustering of large magnitude events occurs, or assess the extent to which clustering localizes strain in a way that produces local seismic hazard above or below the level assessed for the entire region.

Our results suggest that for a transect crossing seven active normal faults, some of which show post-glacial activity^34^, with an across-strike fault spacing of 5–20 km between the three sampled faults, a strain-rate equivalent to 30–75% of the decadal strain-rate from GPS can be accommodated for several millennia by a single fault experiencing an earthquake cluster. We show that such clusters coincide in time with periods of quiescence on faults across strike. We discuss the implications of these findings of out-of-phase behaviour for the regional strain distribution and interpretations of continental deformation processes and seismic hazard.

## Geological background

Central Greece is undergoing continental extension evidenced by historical and instrumental seismicity, satellite geodesy, and active faults that deform Quaternary palaeoshorelines and offset Holocene deposits^34–47^ (Fig. 1). The region around Athens (Attica) is characterized by multiple sub-parallel faults striking ESE -WNW. Fault lengths vary between 9 and 35 km, with relatively low fault slip-rates (0.2–0.5 mm/year) and maximum expected magnitudes up to M 6.6^48,49^. The low slip-rates have produced historical earthquakes, but evidence of surface faulting is sparse, and the region lacks a comprehensive palaeoseismic database^46^ (Fig. 1b). The Fili fault, whose eastern tip could lie beneath Athens (Fig. 1b), may have ruptured in the 1999 Ms 6.0 Athens earthquake that caused 143 deaths and widespread damage in the city, but evidence of primary surface ruptures is debated^50,51^.

## Approach and methods

The Milesi fault, the Malakasa fault and the Fili fault have been sampled for ^36^Cl because they lie on a NNE-SSW transect between two GPS stations crossing Attica in central Greece. The transect lies parallel to the geodetically-derived principal extension strain in Attica^34^ (Fig. 1). We used the ^36^Cl sample site selection criteria of Cowie et al.^27^, which excludes sites prone to exhumation of fault planes by geomorphic processes rather than surface faulting (Supplement S1). We did not sample other faults along this transect as we were unable to find sites respecting such criteria. For each sampling site, we performed detailed geomorphic site characterization to ensure that the sampled fault surfaces were exposed only through tectonic exhumation during repeated earthquakes, as evidenced by horizontal un-incised hanging-wall cut-offs not affected by mass-movement (Supplement S1). We collected multiple discontinuous samples (spacing between samples 23–31 cm, wider on higher and weathered sectors of fault planes) from each fault in the orientation of the fault slip vector. We modelled the slip histories using the Beck et al.^28^ code, which is specifically designed to recover slip-rate variations on faults from discontinuous sampling.

Beck et al.^28^ code models fault slip histories from the ^36^Cl concentrations using a Bayesian MCMC code^28^ (details of the modelling inputs and priors are given in Supplement S2a,b). This code iterates the slip history several million times, forward modelling expected ^36^Cl concentrations each time, and attempting to minimize misfit to measured ^36^Cl concentrations. The code accounts for uncertainty introduced by input parameters and model set-up, and tests for convergence onto a stable result using parallel Markov chains (see Beck et al.^28^ for details). Beck et al.^28^ code iteratively searches for the number of slip events and displacement sizes rather than pre-defining this, and allows the user to concentrate study on identifying periods of time with high or low slip-rate that may be associated with temporal earthquake clustering, rather than individual earthquakes. The code iterates colluvial density, production rates and attenuation lengths associated with spallogenic and muonogenic ^36^Cl production. We allowed the code to propose potential slip histories drawn from a Brownian-passage-time model (BPT) of earthquake recurrence back to 120 ka, which allows for constant or fluctuating pre-Last Glacial Maximum (LGM) slip-rates and associated ^36^Cl production. We allowed ^36^Cl preservation from 80 ka, a time period that allows iteration of the age of the initial ^36^Cl preservation influenced by climate, erosion-rate and slip-rate changes during and prior to the LGM. In other words, we did not force the code to constrain slip histories to occur only after the LGM. We did this for two reasons. (1) We suspected that some fault scarps in the region may record slip histories longer than the time period since the demise of the LGM, a time generally considered to mark the cessation of high erosion during the LGM that works against ^36^Cl preservation^23,27^. We suspected this because Robertson et al.^47^ showed that ^36^Cl exposure ages can be derived from wave-cut platforms carved in limestones at low elevations in central Greece, with preserved geomorphic features suggesting very low erosion rates close to sea-level. We noticed that some fault planes in Attica are at low elevations and are made of similar lithologies to the wave-cut platforms. Hence, these fault planes may have survived erosion during the LGM and part of the preceding glacial period. (2) Initial model runs where earthquake histories started at 30 ka, with preservation allowed from between 12 and 20 ka, could not account for the relatively-high ^36^Cl concentrations measured near the top of the fault planes on some of our sites. To overcome this problem we did not specify an arbitrary value for a time period of pre-exposure to ^36^Cl production, or allow the inference modelling to search for a single value of pre-exposure, or allow a search for a single constant peri-glacial slip-rate to explain pre-exposure, as occurs with other codes^23,52,53^. Instead, as mentioned above, the Beck et al.^28^ code draws potential slip histories from a BPT model of earthquake occurrence, and hence tests ^36^Cl production histories produced by a wide range of constant or fluctuating pre-LGM slip-rates against the measured ^36^Cl concentrations. This approach allows for ^36^Cl production far enough back in time to model growth of the entire scarp. All other priors are as stated in the code published by Beck et al.^28^.

Furthermore, the Beck et al.^28^ code contrasts with some alternative codes because it does not require pre-definition of the number of displacement events, something that cannot be achieved with the relatively-sparse sampled datasets presented in this paper because so-called “cusps” cannot be identified. To counter this, the Beck et al.^28^ code iterates the slip sizes and timings as part of the inference, and it has been shown to be able to resolve slip-rates pulses even with discontinuous sampling (Supplements S3b,c; details in Beck et al.^28^). Finally, our interpretation defines temporal earthquake clusters as periods of rapid slip where the slip accumulated is too large to be accommodated by single earthquakes, defined by the maximum expected coseismic slip for a given fault as per length-displacement scaling.

## Results

### Modelling of fault slip histories from ^36^Cl concentrations

**Figure 2:**
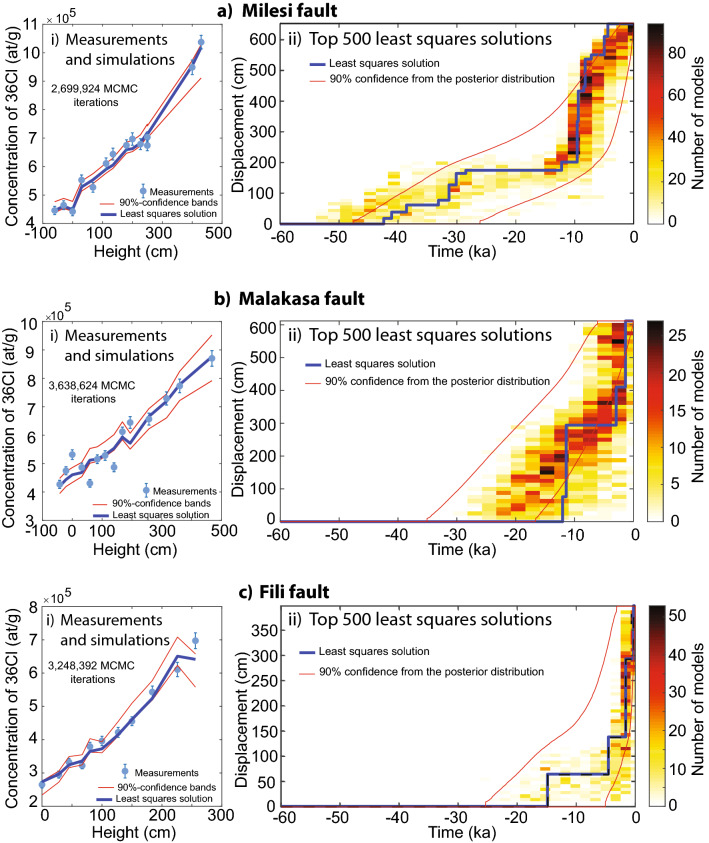
Inference of slip histories from ^36^Cl concentrations measured on the Milesi, Malakasa, and Fili faults (**a**–**c**). (**a.i**–**c.i**) show the measurements and Bayesian posterior distributions of the concentrations of ^36^Cl. (**a.ii**–**c.ii**) show the best 500 least squares solutions for slip histories for the 3 faults. Blue lines are the least squares solutions. Red lines show the 90% confidence interval from the posterior distribution. Full Bayesian solutions are shown in Supplement S2c3.

Concentrations of ^36^Cl increase up the dip and slip vector azimuth of sampled dip-slip fault planes, with some disturbance in this trend produced by variation in the Ca concentration which is accounted for by our MCMC modelling (Fig. 2). We show proposed slip histories via the least squares solution and by an ensemble of the top 500 least squares solutions, as these reveal slip histories that are the best fits to the ^36^Cl data, and are not penalized by prior information we impose in the modelling which are subject to their own uncertainty and therefore they may bias the modelling (See Supplement S2c). More details on the modelling are shown in the Supplements of this manuscript.

### Longevity of recovered slip histories

**Figure 3:**
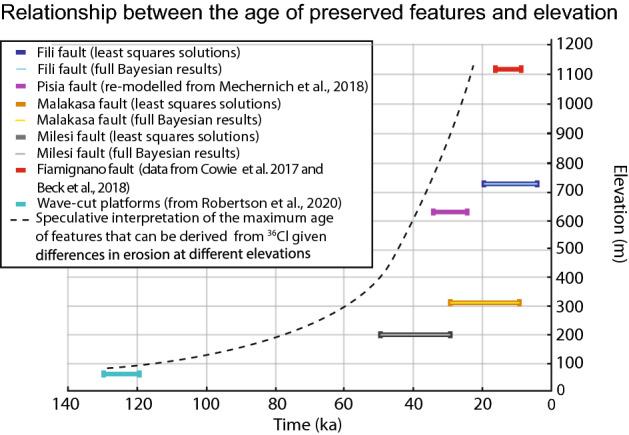
Comparison between the inferred age since the fault scarps started to be preserved, and the elevation of the sampling sites. Together with the three faults subject of study in this paper, we report data from the Fiamignano fault, located in central Italy and studied with ^36^Cl where pre-exposure have not been imposed (Cowie et al.^27^), and re-modelled ^36^Cl data from the Pisia fault plane in central Greece (Mechernich et al.^54^). The bars show the range of possible values reported. We plot the range of possible values from both the least squares solutions (from Fig. 2) and from the highest likelihood solutions (from Supplement S2c). For comparison, we also show the preservation age of ~ 125 ka wave-cut platforms cut into limestones in central Greece, which are at low elevations, and subject to very low erosion rates evidenced by preserved lithophagid borings that were originally only 5–10 cm in depth (Robertson et al.^47^).

The results indicate Holocene slip on all three faults confirming that they should be considered active faults where surface slip has progressively produced growth of the surface fault scarp (Fig. 2). The data also reveal that the recovered history of surface faulting extends back in time by differing amounts on the three faults (Fig. 3). For the Milesi fault scarp the least squares solution suggests an age of ~ 43 ka with the ensemble of least squares solutions suggesting a range of ~ 50–30 ka. For the Malakasa fault scarp the least squares solution suggest an age of ~ 13 ka with the ensemble of least squares solutions suggesting a range of ~ 30–10 ka. For the Fili fault scarp the least squares solution suggest an age of ~ 15 ka with the ensemble of least squares solutions suggesting a range of ~ 20–5 ka. These ages when combined with the slip across the scarps measured using scarp profiles (see Supplement S1) imply slip-rates averaged over the entire scarp age of 0.22–0.13 mm/year (Milesi fault; 6.53 m slip in 30–50 ka, with 0.15 mm/year indicated by the least squares solution), 0.61–0.20 mm/year (Malakasa fault; 6.12 m slip in 10–30 ka, with 0.47 mm/year indicated by the least squares solution), and 0.80–0.20 mm/year (Fili fault; 3.98 m slip in 5–20 ka, with 0.27 mm/year indicated by the least squares solution). To provide a more complete picture of the variability of the preservation ages, we have compared our results with preservation ages obtained from our own re-modelling of the Pisia fault^54^ (Central Greece) using the Beck code, and ages from the Fiamignano fault in Central Italy, already modelled with the Beck code^27,28^ (Fig. 3; see Supplement S3a). We only plot results from these two sites because their modelling does not include arbitrary pre-exposure values, a search for a single pre-exposure value during the inference, or a search for a constant peri-glacial slip-rate in the inference. The results appear to confirm our suspicion that older scarps may be preserved at low elevations. This result is interesting because it opens the possibility of gaining very long slip-histories for low elevation sites with low erosion rates during the last glacial, introducing the possibility of scarps surviving from before the LGM.

### Slip-rate histories

**Figure 4:**
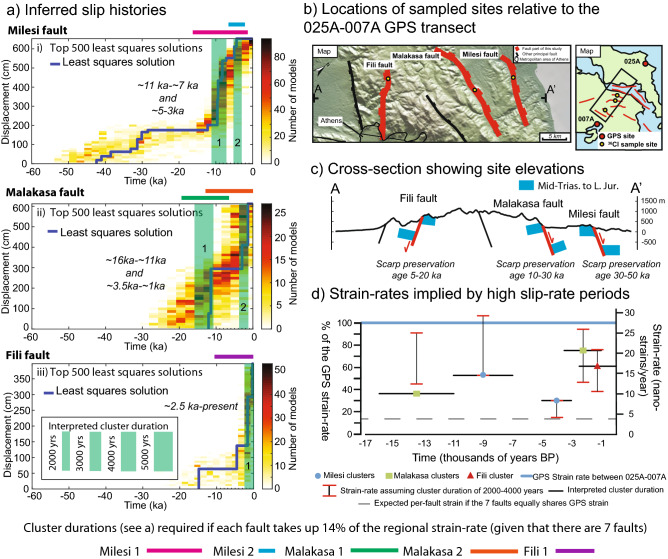
Swapping of the location of fault activity/strain accumulation across the strike of the normal fault system. We concentrate on the time since ~ 15 ka because slip is recorded at all 3 sites during this time period. (**a**) Slip-histories inferred from ^36^Cl data interpreted to show the approximate durations of the principal clusters. (**b**) Locations of the faults relative to the 025A-007A GPS transect. (**c**) Cross-section showing the elevations and preservation ages of each site and geomorphic and geological offsets. (**d**) Strain rates implied by high slip-rate periods; strain-rates in each cluster are calculated over the interpreted duration of the cluster, as shown in (**a**); uncertainty for each strain-rate value is calculated assuming a cluster duration of 2000 kyears and 4000 kyears. We reject the hypothesis that each of the seven principal faults slip at a constant rate taking up 1/7 (~ 14%) of the regional strain-rate because the required clustered durations are not consistent with the inferred least squares slip histories. We interpret the existence of earthquake clusters that are out-of-phase on faults located across from each other suggesting that only a few faults are active at any one time on a millennial timescale and fault activity swaps across strike to maintain the regional strain-rate.

The existence of (1) fault-controlled topographic escarpments for the three faults, with changes in elevation of hundreds of metres (Fig. 4c), and (2) offsets of bedrock pre-rift strata ranging between ~ 600 m and ~ 1200 m^46,51^ suggest that faulting on the Milesi, Malakasa and Fili faults was underway long before ~ 30–50 ka, ~ 10–30 ka and ~ 5–20 ka, respectively (see geological cross-sections across the Malakasa and Fili faults in Supplement S2d). If the slip-rates averaged over the entire scarp ages derived from the least squares solutions are applied and kept constant back through time, the kilometric offsets would have taken several million years to develop (Supplement S2e), consistent with proposed ages for extension in the region^38,43^. Thus, it is clear that our ^36^Cl data only recover the most recent part of the slip history, with the isotopic evidence for early slip removed by earlier erosion.

The analysis of the slip histories suggests that it is hardly possible to interpret the ^36^Cl as implying constant slip-rates for the 3 faults since ~ 30–50 ka, ~ 10–30 ka and ~ 5–20 ka. Instead, concentrating on the time since ~ 15 ka where we have results from all 3 sites, we suggest that the faults experienced episodic behavior, with alternating periods of rapid slip accumulation and quiescence (Figs. 2 and 4). Periods of rapid slip on one fault are synchronous with periods of quiescence on faults across strike (Fig. 4). For the Milesi scarp our result suggest pulses of rapid slip accumulation at ~ 30–40 ka, ~ 11–7 ka and ~ 5–3 ka (Fig. 2a,ii). A short period of quiescence is suggested to occur between the two younger clusters on the Milesi fault, as marked by the lack of slip accumulation shown by both the least squares solution and the ensemble of least squares solutions in that time interval. Moreover, the ensemble of least squares solutions for the Milesi fault suggests ~ 1.2 m of surface slip between ~ 5–1 ka, and this is comparable to the ~ 1.8 m of surface offset since 4 ka measured through palaeoseismic trenching^46^. For the Malakasa scarp our results suggest pulses of rapid slip accumulation at ~ 16–11 ka and ~ 3.5–1.0 ka (Fig. 2b,ii). For the Fili scarp our results suggest a pulse of rapid slip accumulation at ~ 2 ka to the present day (Fig. 2c,ii).

## Discussion

### Assessment of the temporal constraints achievable with discontinuous sampling

To assess the temporal constraints achievable with discontinuous sampling we compared our results with those from semi-continuous sampling of fault planes with the re-modelled data from the Pisia fault and data from the Fiamignano fault (Supplement S3a). Both of these faults were sampled with semi-continuous sampling, in contrast to the wider sampling we achieved on the faults presented in this study. Firstly, we confirm that data from semi-continuously sampled faults imply changes in the rate of slip through time (Supplement S3a). Secondly, results gained using different codes produce comparable results, for example the same high slip-rate pulse at ~ 10–7 ka is identified using the codes from Schlagenhauf et al.^23^, Cowie et al.^27^ and Beck et al.^28^, although we are wary of the result using the Schlagenhauf et al.^23^ code as it uses pre-exposure (Supplement S3b). Thirdly, we show that the Beck code is able to resolve the high slip-rate pulse at ~ 10–7 ka even with discontinuous sampling, because the results using the full Pisia dataset are very similar to those from a degraded datasets where 50% and then 75% of the samples are artificially removed during the modelling (Supplement S3c). We also show that the values we used whilst running the Beck code (120 ka for the start of the model run and 80 ka the initiation of when ^36^Cl can be generated) are sensible and do not bias the results because lower values (100 ka and 60 ka) produce very similar results (Supplement S3c). We conclude that the sample spacings we achieved for the Fili, Milesi and Malakasa faults, and values we used for the time parameters for ^36^Cl production and preservation during the run, are sufficient to resolve slip-rate variability and the existence and timings of clusters lasting several millennia.

### Implications on fault-based seismic hazard assessments

Our modelling suggests that slip-rate pulses are characterised by 1.0–3.5 m of slip (Figs. 2, 3, 4; Supplement S4). We interpret these as temporal earthquake clusters because the faults are too short in length to experience coseismic slip of these amounts in single earthquakes. For example, Deligiannakis et al.^49^ suggests maximum slip magnitudes of 0.32 m, 0.80 m and 0.52 m respectively for the Milesi, Malakasa and Fili faults, calculated using scaling relationships established by Wells and Coppersmith^55^. The same is true if we use quantitative scaling relationships provided by Pavlides and Caputo^56^ and Wesnousky^57^. Thus, we interpret these 1.0–3.5 m slip pulses as temporal earthquake clusters, alternating with periods of quiescence (i.e. temporal earthquake anti-clusters). Furthermore, examination of the slip since 15 ka shows that periods of rapid slip and quiescence alternate through time on the three faults, defining out-of-phase slip periods on faults located across strike (Figs. 2, 3, 4). The clusters on different faults tend not to overlap in time, with activity swapping rapidly across strike as a cluster terminates.

Our findings of alternating clusters on parallel faults provide insights in understanding how earthquakes are distributed across faults within normal fault systems, and how these information can impact on fault-based seismic hazard assessments. In fact, although it has been suggested that earthquake activity is clustered in space and time and can migrate across strike between parallel sets of active faults over a variety of timescales^25,58–61^, prior to our paper data on continuous records of earthquake clustering encompassing multiple seismic cycles on faults located across-strike were scarce. Therefore, it was challenging to incorporate such information into seismic hazard assessment. This paper provides new pertinent observations that help in the latter: (1) Earthquake clusters swap both into the hangingwall and footwall of faults spaced at least 5–20 km across strike; (2) clusters may begin on faults within the first thousand years after activity ceases on faults across strike; (3) Clusters last several millennia; (4) Clusters can involve 2.0–3.5 m of slip which is 78–100% of the slip measured over 15 millennia; (5) Slip-rates and earthquake recurrence intervals within clusters increase by about 3–4 times compared to that calculated since 15 ka (Supplement S5). These observations have implications for probabilistic assessments of the occurrence of the next earthquake, because whether an earthquake history is clustered or not can be more important than the probability density function chosen to describe the recurrence times, with the resultant probability dependent on whether a cluster is treated as ongoing or over^62^. Our study shows that faults near Athens, although characterized by low slip-rates, are active and capable of releasing surface-rupturing earthquakes, and given the uncertainty of whether clusters and anti-clusters are ongoing or about to end, the recognition of clustered fault activity should be included in future probabilistic seismic hazard assessments for the city.

### Implications on geodetic measurements of regional deformation

Our observations of spatial and temporal earthquake clustering raise the question of how extension rates on individual faults relate to the extension rate across the entire region. In fact, the observation of out-of-phase clustering between parallel faults suggests that not every fault contributes in the same amount to the regional deformation at the same time. Previous studies have attempted to use decadal geodetic horizontal strain-rates to resolve individual strain-rate contributions from post-glacial slip-rates of multiple faults in Attica, including faults we have sampled^34^. However, post-glacial slip-rates do not provide sufficient time resolution to resolve earthquake clustering lasting only a few millennia during the Holocene. Hence, it is unclear how earthquake clustering impacts calculations of individual strain-rate contribution of single faults.

To explore this, we have calculated and compared the horizontal geodetic strain-rate measured between two permanent GPS stations (007A-0025A) aligned on the transect containing the ^36^Cl sites and the strain-rates associated with each cluster observed in the faults slip histories (Figs. 1a and 4). Strain-rates inferred from the ^36^Cl data on faults associated with clusters are calculated assuming a 45º dip angle for the faults, similar to those we measured at the surface, and dip-slip motion. Strain-rates are calculated over the interpreted duration of clusters, and uncertainty is assessed by assuming variable cluster duration between 2000 and 4000 ka. We assume that the decadal GPS strain-rate applies over longer time periods for the purpose of our comparison. The decadal horizontal strain-rate between GPS stations 025A and 007A from Chousianitis et al. (2013) is 27 × 10^–9^ year^−1^. The preferred heave-rate implied by slip during individual clusters equates to a strain-rate of between 8.34 × 10^–9^ year^−1^ and 20.69 × 10^–9^ year^−1^, calculated over the horizontal distance between stations 025A and 007A of 78 km (Fig. 4; Supplement S4). These values are a relatively large percentage (30–75%) of the regional strain-rate if the GPS rate applies over longer time scales. To achieve values as low as ~ 14% of the regional strain-rate, that is the value expected if all the 7 principal faults on the transect accommodate equal shares of the extension and slip at constant rates, the slip could not exhibit spatial and temporal clustering. This is because cluster durations would have to be on the order of 10.8–15.0 kyears in duration for the 4 clusters with slip > 2.5 m, and they would have to overlap in time, with deformation that would not display obvious clustering. Moreover, constant slip-rates through time are not supported by the least squares solutions or posterior distribution from the ^36^Cl modelling (Fig. 4); we reject this possibility. Note that we are not aiming to constrain the complete record of the spatial distribution of palaeoearthquakes in Attica, for which we would have needed to sample all of the faults across the transect, something that we were not able to do as we could not found suitable ^36^Cl sampling sites on some faults. However, our data show that, assuming the cluster durations we derive are reasonable, individual faults can accommodate 30–75% of the regional extension (assuming the GPS rate reflects the Holocene rate), suggesting that only a small number of the active faults on an across-strike transect contribute to the regional strain-rate at any given time. The implication is that during the clusters, earthquake recurrence intervals for given magnitudes would be about 3–4 times shorter than if strain were uniformly spread across the region (Supplement S5).

These findings have implications for how one should interpret geodetic results. Geodesy is used to study seismic hazard and continental deformation, but debate remains on how to interpret such data^18,63–67^. Our results show that: (1) The implied strain-rates during measured earthquake clusters do not exceed the strain-rate implied by decadal measurements with GPS, suggesting that GPS-derived strain-rates can be representative for the long-term regional deformation rate; (2) If several active faults exist between a pair of GPS stations, it will not be possible to resolve whether strain is concentrated on one fault or shared between all faults or how strain localization changed through the Holocene solely using the GPS data. Therefore, we suggest that the linkage between ^36^Cl dating (and possibly other palaeoseismic approaches) and geodetic data is a powerful tool to study continental deformation and seismic hazard because it combines the long and short-term views of the deformation. That is because ^36^Cl offers the possibility to obtain continuous records containing tens of millennia of activity on normal faults, something that it is not always possible to gain from traditional trenching. Therefore, we advocate dense GPS networks with stations on every fault block combined with InSAR observations that provide continuous spatial coverage of strain accumulation if the precise location and width of actively deforming zones is required, alongside palaeoseismology covering many millennia, for example ^36^Cl studies, where possible. This approach can impact on seismic hazard assessments, because it allows us to discretize how the probability of ruptures is distributed across a fault system, information that is not obtainable by using an approach based only on the strain-release distribution gained by widely-spaced GPS stations.

We are aware that ^36^Cl exposure histories can only be gained if the fault planes are well-exposed and well-preserved, so this approach will not be suitable for all deforming regions, but they can give information from well-exposed fault systems that can be used to guide interpretations of less well-exposed regions. Although our results refer to normal faulting, it is perhaps likely that the same pattern of strain release through localized clustering may apply to brittle continental deformation in general, affecting therefore also thrust and strike-slip faulting.

We are aware that there is microseismicity widespread across the crustal volume of Attica, some of which may be associated to faults with surface offsets and some distributed between the obvious faults at the surface^34^. We suggest that this is perhaps the “distributed sources” of seismicity that are envisaged following observations that major faults are surrounded by many minor faults, and that there is a Gutenberg-Richter magnitude-frequency distribution of seismicity in a region^68^. Following studies showing that the majority of strain is accommodated by slip along the largest faults^69,70^, we add a qualifier to our conclusions because we can only constrain strain accumulation on major faults that produce surface faulting with ^36^Cl data. Thus, we conclude that our results apply to the major faults and the strain contribution from “distributed sources” needs further study.

## Conclusions

The opportunity to combine geodetic measurements of regional strain-rate over decades and fault slip histories from ^36^Cl covering many millennia provide a powerful combination to study continental deformation and seismic hazard. This study suggests that earthquake slip is clustered in time, with out-of-phase clustered activity on faults located across strike from each other. Over many millennia strain accumulates across the entire width of the fault system containing numerous faults, but individual faults are actively-slipping at different times for time periods lasting only a few millennia. Existing geodetic data alone cannot resolve the swapping slip or that slip in particular locations is clustered in time. The results suggest that denser geodetic observations will help improve the spatial resolution of active strain-accumulation pertinent to seismic hazard calculations and continental deformation, but palaeoseismic data such as that provided by ^36^Cl studies are needed to define the variability through time of earthquake recurrence that occurs over several millennia.

## Methods

### Attributes of sampling sites and samples collection

Sampling sites have been chosen distant from drainage incisions, have planar, un-eroded upper and lower slopes, and have horizontal and parallel hangingwall and footwall cut-offs; this implies exhumation solely due to fault slip. The UTM locations of the sampling sites are: Milesi fault 34 S 38.28534 N 23.78163 E; Malakasa fault 34 S 38.23590 N 23.74945 S; Fili fault 34 S 38.14880 N 23.63759 E. The sampling sites are characterized by planar fault planes, with constant dip, and preserved mm-scale striae, proving minimal erosion of the fault planes since surface exposure. Scarp profiles were constructed perpendicular to the strike of the fault using a hand-held laser range finder, in order to constrain key parameters for modelling the slip histories, such as the values of dip of the upper slope, the fault dip, the dip of the lower slope and the on-fault slip. Fault samples were collected with spacing between 23 and 31 cm up the fault plane in the vertical plane containing the slip vector, but with some gaps where fault plane preservation was poor. Structural data were collected to determine the fault orientation and slip vector using a compass clinometer. Where the fault plane showed weathering, samples were selected to avoid such areas, but to maintain the pattern of sample collection at successively higher positions on the fault plane. To collect samples below the surface, providing extra information for modelling of the most recent earthquakes, trenches up to 0.6 m deep have been dug in front of the fault planes. Samples have dimensions (length, width) of 15 × 5 cm and thickness of 2.5 cm. Samples were prepared following the procedure outlined in Schimmelpfennig et al. (2009), and analysed with AMS at SUERC, Scotland, to determine the concentrations of ^36^Cl in each sample.

### Modelling of ^36^Cl data

The ^36^Cl concentrations of the samples were modelled using a flexible Bayesian Reverse Jump Markov-chain Monte Carlo (MCMC) algorithm, published by Beck et al.^28^ to infer the fault slip histories. The code searches for the highest likelihood and least-squares solutions to the measured data iterating key parameters such as the magnitude and timings of slip events, the density of the colluvium, the ^36^Cl production factors and the timing of ^36^Cl initial production. The code tabulates all the possible radiation geometries before the modelling starts, and it draws from this table during each iteration, rather than calculating it each time. Convergence during the modelling, with parallel Markov chains, is tested with a Gelman-Rubin test. The full approach to modelling the ^36^Cl data is described in Beck et al.^28^. We choose to display slip histories via both the least squares solution and by an ensemble of least squares solutions, as these reveal slip histories that are the best fits to the ^36^Cl data, and are not penalized by prior information we impose in the modeling. We chose this approach as although priors are relatively well-known (spallogenic and muonogenic ^36^Cl production rates and attenuation lengths for given latitude and elevations; rock and colluvial densities; timings of changes in erosion rate; amounts of slip per event; etc.), we feel that the least squares solutions help to confirm or deny our preferred values for priors, helping us to recognise any bias we have imposed in our choice of priors. Our results show that, in any case, the least squares solutions produce results that fall within the bounds of the priors we have chosen, suggesting they are sensible. We also show Bayesian highest likelihood solutions from the posterior distributions in Supplements S2c and S3c for completeness. A maximum slip per event between 10 and 300 cm is explored by the code we use, values up to a factor of 6 higher than that expected for faults of these lengths (e.g. Wells and Coppersmith^55^). The composition of each rock sample is included in the modelling, such as the concentrations of major elements expressed in oxides (Al_2_O_3_, Fe_2_O_3_, MnO, MgO, CaO, NA_2_O, K_2_O, TiO_2_, P_2_O_5_), the natural content of ^35^Cl, the content in ^40^Ca, the concentration of ^36^Cl AMS and the uncertainty in ^36^Cl values. The composition of the colluvium is taken from the average chemical composition of samples of colluvium collected in central Italy, where the climate conditions and the parental rocks are similar to central Greece (as per Cowie et al.^27^). Our modelling shows that the range of iteration of the production rates and colluvial densities for the actual run is larger than the variation in these parameters implied by differences in the colluvial composition, implying that variations in colluvial composition do not significantly affect the modelling of fault slip histories (Supplement S6). Values of latitude and elevation of each field site have been used to calculate scaling factors for spallation and muon capture, following Stone et al. (2000), although these values are iterated by the code. A burn in of 50% has been applied to the modelling of each fault to de-emphasise initial results produced before the MCMC code has converged.

## Supplementary Information

Supplementary Information 1.

Supplementary Information 2.

Supplementary Information 3.

Supplementary Information 4.

Supplementary Information 5.

Supplementary Information 6.

Supplementary Information 7.

Supplementary Information 8.

Supplementary Information 9.

Supplementary Information 10.

Supplementary Information 11.

Supplementary Information 12.

Supplementary Information 13.

Supplementary Information 14.

Supplementary Information 15.

Supplementary Information 16.
