# Supplementary material for: Temporal and spatial earthquake clustering revealed through comparison of millennial strain-rates from 36Cl cosmogenic exposure dating and decadal GPS strain-rate
Source: Sci Rep. 2021 Dec 2;11:23320. doi: 10.1038/s41598-021-02131-3 (PMC8639784; doi:10.1038/s41598-021-02131-3)
Supplement: Supplementary file 1 — Supplementary Information 1. [file 41598_2021_2131_MOESM1_ESM.docx]

**Supplemental material for:**

**Temporal and spatial earthquake clustering near Athens, Greece, revealed through comparison of millennial strain-rates measured with ^36^Cl cosmogenic exposure dating and decadal GPS strain-rate.**

Francesco Iezzi^1, 2^, Gerald Roberts^2^, Joanna Faure Walker^3^, Ioannis Papanikolaou^4^, Athanassios Ganas^5^, Georgios Deligiannakis^4^, Joakim Beck^6^, Soeren Wolfers^6^, D. Gheorghiu^7^

*^1^* *DiSPUTer, Università degli Studi “Gabriele d’Annunzio” Chieti-Pescara, Via dei Vestini, Chieti, 66100, Italy*

^2^ *Department of Earth and Planetary Sciences, Birkbeck, University of London, Malet Street, London, WC1E 7HX, UK*

*^3^ Institute for Risk and Disaster Reduction, University College London, Gower Street, London, WC1E 6BT, UK Athens, Greece*

*^4^ Mineralogy-Geology Laboratory, Department of Natural Resources Development and Agricultural Engineering, Agricultural University of Athens, 75 Iera Odos, 118-55*

*^5^ Institute of Geodynamics, National Observatory of Athens, Lofos Nymfon, 11810 Athens, Greece*

*^6^Computer, Electrical and Mathematical Sciences & Engineering (CEMSE), King Abdullah University of Science and Technology (KAUST), Thuwal 23955-6900, KSA*

*^7^ Scottish Universities Environmental Research Centre*, *Rankine Avenue, East Kilbride, G750QF, UK*

The supplemental material is subdivided in 6 parts.

**Supplement S1: Site characterization of ^36^Cl sampling sites.** This supplement presents location maps, photographs and scarp profiles for each of the three faults sampled indicating that the sampled fault planes were exhumed by tectonic activity and not erosion.

**Supplement S2: Parameters used to model the measured ^36^Cl concentrations on the fault planes using the Beck et al. (2018) code and Bayesian results.** This supplement is subdivided in:

- **Supplement S2a**: Calculations for deriving values applied in the Mag_field file, necessary to correct the ^36^Cl production for the latitude and elevation of the sampling site;
- **Supplement S2b**: Input files for modelling of ^36^Cl concentrations with the Beck et al. (2018) code. The “m file” document presents data used as input for the Matlab file (**Supplement S2b.i**). The “rock file” document presents the concentration of ^36^Cl and the elemental composition data for the three faults (**Supplement S2b.ii**). The “colluvium file” presents the elemental composition of the colluvium, calculated as the average of a set of measurements of previously published data in Central Italy, where climatic conditions and parental rocks are similar (from Cowie et al., 2017) (**Supplement S2b.iii**). The “mag_field file” presents the scaling factor for the spallation and muons capture given the altitude and the latitude of the sampling sites (from Stone et al., 1998) (**Supplement S2b.iv**).
- **Supplement S2c**: Full Bayesian results using the input data in Supplement S2b.
- **Supplement S2d**: New geological cross-sections across the Fili and Malakasa faults used to measure the geological throw between pre-rift strata on both the hangingwall and footwall of the faults.
- **Supplement S2e**: Calculations of fault slip rates over the entire scarp age, as obtained from 36Cl modelling.

**Supplement S3: Re-modelling of Fiamignano and Pisia faults.** This supplement is subdivided in:

- **Supplement S3a**: Re-modelling of pre-existing datasets of ^36^Cl measurements on the Fiamignano fault (Cowie al., 2017) and Pisia fault (Mechernich et al., 2018) using the Beck et al. (2018) code
- **Supplement S3b**: Comparison of Schlagenhauf, Cowie and Beck codes. It shows that the application of different modelling approaches can resolve the same slip rate pulses on the Pisia fault.
- **Supplement S3c**: Comparison of runs varying sample density and initial 36Cl production choices. It shows that the variation of initial ^36^Cl production and the density of the samples do not affect the modelling of the slip history.

**Supplement S4: Strain-rate calculations.** It contains the spreadsheet with the calculations of the strain-rates measured with GPS from Chousianitis et al. (2013) and those inferred from the ^36^Cl data. Please note that similar values of strain are presented in newly published papers: (1) P. Briole, A. Ganas, P. Elias, D. Dimitrov, The GPS velocity field of the Aegean. New observations, contribution of the earthquakes, crustal blocks model, Geophysical Journal International, Volume 226, Issue 1, July 2021, Pages 468–492, <https://doi.org/10.1093/gji/ggab089>; (2) N. D'Agostino, M. Métois, R. Koci, L. Duni, N. Kuka, A. Ganas, I. Georgiev, F. Jouanne, N. Kaludjerovic, R. Kandić, Active crustal deformation and rotations in the southwestern Balkans from continuous GPS measurements, Earth and Planetary Science Letters, Volume 539, 2020, 116246, ISSN 0012-821X,

**Supplement S5: Fault behaviour.** This supplement compares throw-rates and earthquake recurrence intervals implied by deformation rates averaged since 15 ka, and those within individual earthquake clusters.

**Supplement S6: Variability in colluvium elemental composition and its effect on the ^36^Cl production rates.** This supplement contains calculations to show that the range of iteration of the production rates and colluvial densities for the Fili model run is larger than the variation in these parameters given by iteration of the elemental composition of the colluvium. This implies that the uncertainty in colluvial composition does not significantly affect the modelling of fault slip histories.
