# Supplementary material for: Temporal and spatial earthquake clustering revealed through comparison of millennial strain-rates from 36Cl cosmogenic exposure dating and decadal GPS strain-rate
Source: Sci Rep. 2021 Dec 2;11:23320. doi: 10.1038/s41598-021-02131-3 (PMC8639784; doi:10.1038/s41598-021-02131-3)
Supplement: Supplementary file 2 — Supplementary Information 2. [file 41598_2021_2131_MOESM2_ESM.pdf]

SUPPLEMENTAL MATERIAL 1

Sampling sites characterization and fault sampling approach

The following figures show the characterizations of the sampling sites for the three faults. The sampling sites are characterized by planar fault planes, with constant dip, and preserved mm-scale striae, proving minimal erosion of the fault planes through time. Fault samples were collected every ~30 cm up the fault plane in the vertical plane containing the slip vector. Samples have dimensions of 15 x 5 cm and thickness of 2.5 cm. Details on the approach followed in the selection of the suitable sampling site and in the collection of samples from the fault plane are outlined in the Methods section within the main text.

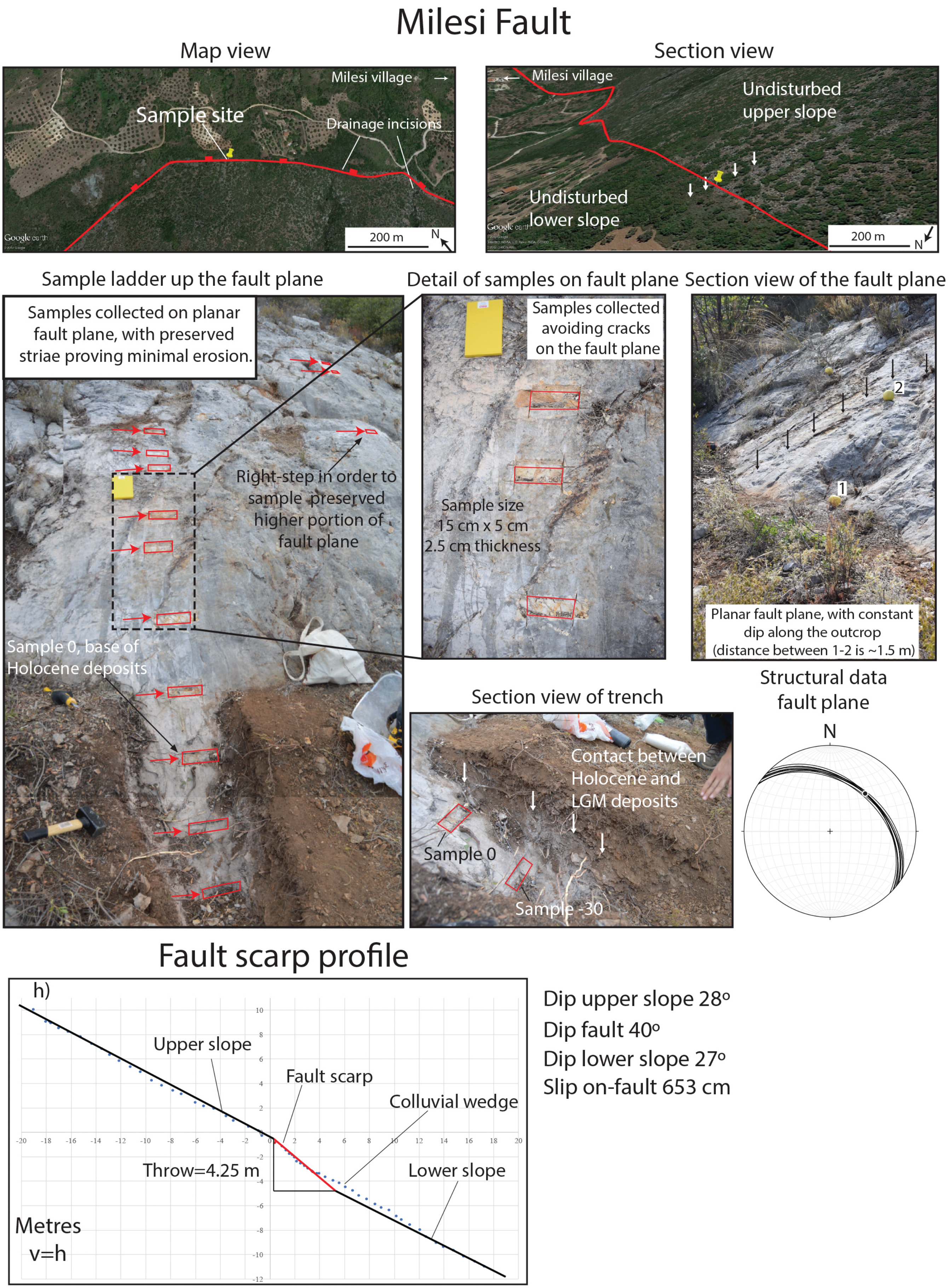
Milesi Fault
Map view
Milesi village →
Sample site
Drainage incisions
200 m
N
Section view
Milesi village
Undisturbed upper slope
Undisturbed lower slope
200 m
N
Sample ladder up the fault plane
Samples collected on planar fault plane, with preserved striae proving minimal erosion.
Right-step in order to sample preserved higher portion of fault plane
Sample 0, base of Holocene deposits
Detail of samples on fault plane
Samples collected avoiding cracks on the fault plane
Sample size 15 cm x 5 cm 2.5 cm thickness
Section view of the fault plane
1
2
Planar fault plane, with constant dip along the outcrop (distance between 1-2 is ~1.5 m)
Section view of trench
Contact between Holocene and LGM deposits
Sample 0
Sample -30
Structural data fault plane
N
Fault scarp profile
h)
Upper slope
Fault scarp
Colluvial wedge
Throw=4.25 m
Lower slope
Metres
v=h
Dip upper slope 28°
Dip fault 40°
Dip lower slope 27°
Slip on-fault 653 cm

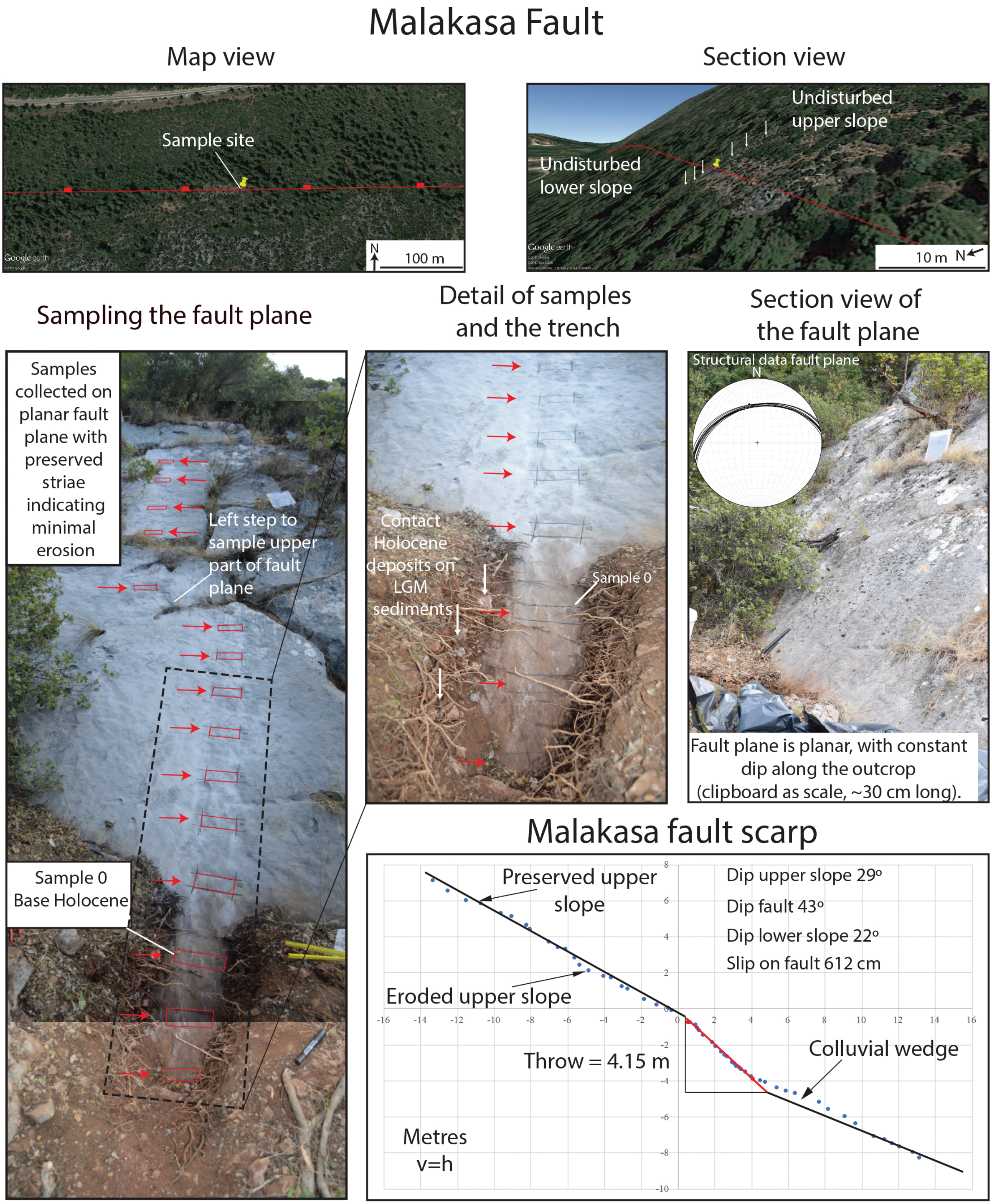

Malakasa Fault
Map view
Sample site
N
100 m
Google earth
Section view
Undisturbed upper slope
Undisturbed lower slope
10 m
N
Google earth
Sampling the fault plane
Samples collected on planar fault plane with preserved striae indicating minimal erosion
Left step to sample upper part of fault plane
Sample 0 Base Holocene
Detail of samples and the trench
Contact Holocene deposits on LGM sediments
Sample 0
Section view of the fault plane
Structural data fault plane
N
Fault plane is planar, with constant dip along the outcrop (clipboard as scale, ~30 cm long).
Malakasa fault scarp
Preserved upper slope
Eroded upper slope
Dip upper slope 29°
Dip fault 43°
Dip lower slope 22°
Slip on fault 612 cm
Throw = 4.15 m
Colluvial wedge
Metres
v=h

Fili Fault

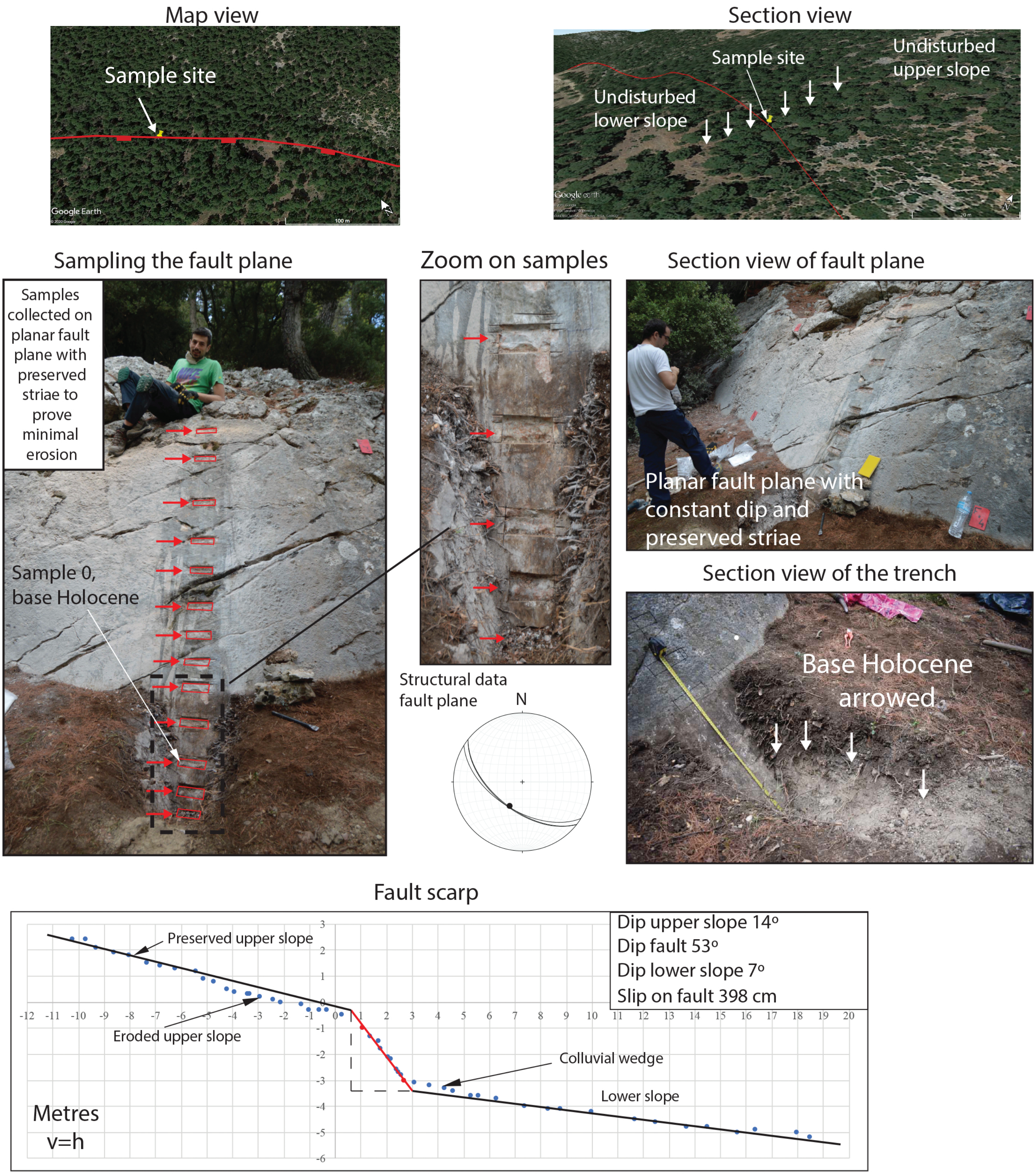

Map view
Sample site
Google Earth
100 m
Section view
Sample site
Undisturbed upper slope
Undisturbed lower slope
Google earth
20 m
Sampling the fault plane
Samples collected on planar fault plane with preserved striae to prove minimal erosion
Sample 0, base Holocene
Zoom on samples
Structural data fault plane
N
Section view of fault plane
Planar fault plane with constant dip and preserved striae
Section view of the trench
Base Holocene arrowed
Fault scarp
Dip upper slope 14º
Dip fault 53º
Dip lower slope 7º
Slip on fault 398 cm
Preserved upper slope
Eroded upper slope
Colluvial wedge
Lower slope
Metres
v=h
-12 -11 -10 -9 -8 -7 -6 -5 -4 -3 -2 -1 0 1 2 3 4 5 6 7 8 9 10 11 12 13 14 15 16 17 18 19 20
3 2 1 0 -1 -2 -3 -4 -5 -6
