# Supplementary material for: Temporal and spatial earthquake clustering revealed through comparison of millennial strain-rates from 36Cl cosmogenic exposure dating and decadal GPS strain-rate
Source: Sci Rep. 2021 Dec 2;11:23320. doi: 10.1038/s41598-021-02131-3 (PMC8639784; doi:10.1038/s41598-021-02131-3)
Supplement: Supplementary file 4 — Supplementary Information 4. [file 41598_2021_2131_MOESM4_ESM.pdf]

SUPPLEMENT S2b.i

Input values used within the “m.file” in the Beck et al. (2018) Matlab code

Fault name	Milesi	Malakasa	Fili
On-fault slip (m)	6.53	6.12	3.98
Dip upper slope (º)	28	29	14
Dip lower slope (º)	27	22	7
Dip fault plane (º)	40	43	53
Trench depth (m)	0.6	0.4	0.2
Rock mean density (kg/m^3)	2.7	2.7	2.7
Max slip per event (cm)	300	300	300
Time window of analysis (ka)	120	120	120
