# Supplementary material for: Temporal and spatial earthquake clustering revealed through comparison of millennial strain-rates from 36Cl cosmogenic exposure dating and decadal GPS strain-rate
Source: Sci Rep. 2021 Dec 2;11:23320. doi: 10.1038/s41598-021-02131-3 (PMC8639784; doi:10.1038/s41598-021-02131-3)
Supplement: Supplementary file 5 — Supplementary Information 5. [file 41598_2021_2131_MOESM5_ESM.pdf]

SUPPLEMENT S2b_ii

Chemical composition of each sample, together with the concentration of ^{36}Cl AMS and its uncertainty.

SITE ALL ALL DATA	Al2O3 (Al)	Fe2O3 (Fe)	MnO (Mn)	MgO (Mg)	CaO (Ca)	Na2O(Na)	K2O(K)	TiO2(Ti)	P2O5(P)	Cl nat AMS	Ca ICP	Z (position on the scarp)	Sample Thickness	[36Cl] AMS	uncertainty
sample name	%	%	%	%	%	%	%	%	%	ppm	ppm	cm	cm	at/g.rock	
MIL-60	0	0.10145316	0	0.43902269	53.3797104	0	0	0	0	2.57463	381504.794	0	2.5	445818.27	14320.10
MIL-30	0	0	0	0.43214121	51.3373988	0	0	0	0	3.07598	366908.393	30	2.5	464181.66	15360.40
MIL0	0	0	0	0.35467603	44.9824461	0	0	0	0	2.22585	321489.546	60	2.5	441995.67	14692.05
MIL+30	0	0	0	0.52703699	53.7549379	0	0	0	0	2.54199	384186.545	90	2.5	552784.94	17750.47
MIL+68	0	0	0	0.52010061	52.6495776	0	0	0	0	3.06967	376286.535	128	2.5	527188.32	17203.62
MIL+112	0	0	0	0.49553249	52.3205755	0	0	0	0	2.49195	373935.157	172	2.5	611027.85	19318.53
MIL+135	0	0	0	0.51920156	51.7042785	0	0	0	0	3.08887	369530.482	195	2.5	643802.35	20840.53
MIL+180	0	0	0	0.54249985	53.1577989	0	0	0	0	3.57466	379918.793	240	2.5	674904.76	18997.03
MIL+200	0	0	0	0.50464754	51.3898152	0	0	0	0	2.49847	367283.013	260	2.5	696791.87	21634.21
MIL+227	0	0	0	0.52003221	50.572312	0	0	0	0	2.72572	361440.317	287	2.5	678821.54	19183.56
MIL+250E	0	0	0	0.4577516	51.7332524	0	0	0	0	2.88196	369737.559	310	2.5	703334.98	16370.76
MIL+250W	0	0	0	0.47033236	51.1725875	0	0	0	0	2.07107	365730.487	310	2.5	674125.25	18519.26
MIL+400	0	0	0	0.51399977	53.8013712	0	0	0	0	1.49406	384518.404	460	2.5	948701.74	25687.27
MIL+430	0	0	0	0.5044822	54.3927748	0	0	0	0	2.67026	388745.165	490	2.5	1037229.48	24053.90
MAL-42	0	0	0	0.54637006	51.0692048	0	0	0	0	3.55291	364991.611	0	2.5	426952.93	14285.02
MAL-21	0	0	0	0.52882232	52.4539153	0	0	0	0	2.61387	374888.137	21	2.5	474685.55	14194.86
MAL0	0	0	0	0.58461642	51.3885159	0	0	0	0	3.89188	367273.727	42	2.5	531688.90	16912.60
MAL+59	0	0	0	0.70917021	53.9608394	0	0	0	0	3.72434	370755.373	74	2.5	486334.94	15443.73
MAL+84	0	0	0	0.56039256	52.3102542	0	0	0	0	2.63471	385658.123	101	2.5	430580.40	13871.99
MAL+110	0	0	0	0.49093068	51.997371	0	0	0	0	4.05550	373861.39	126	2.5	515220.16	15354.04
MAL+140	0	0	0	0.58404585	51.9401756	0	0	0	0	4.20545	371625.215	152	2.5	528503.25	16846.72
MAL+32	0	0	0	0.50596996	51.8756639	0	0	0	0	1.93865	371216.439	182	2.5	486987.88	15459.31
MAL+168	0	0	0	0.4672309	54.3098528	0	0	0	0	3.77020	388152.522	210	2.5	612188.87	15888.60
MAL+194	0	0	0	0.50096958	46.3444861	0	0	0	0	5.85031	331224.046	236	2.5	644462.13	20731.43
MAL+257	0	0	0	0.53904209	49.9087885	0	0	0	0	5.15461	356698.115	299	2.5	656592.98	20710.26
MAL+316	0	0	0	1.04771529	50.0415969	0	0	0	0	3.13045	357647.297	358	2.5	729186.60	22988.94
MAL+360	0	0	0	0.47364402	53.281421	0	0	0	0	1.91738	380802.32	402	2.5	773230.50	24215.91
MAL+420	0	0	0	0.57735963	55.1156466	0	0	0	0	0.25882	393911.53	462	2.5	525424.66	16798.61
MAL+468	0	0	0	0.55455883	54.1998651	0	0	0	0	3.82750	387366.439	510	2.5	870341.68	27479.40
FIL-20	0	0	0	1.26542831	60.8864359	0	0	0	0	10.68996	435155.361	0	2.5	264700.432	9281.31
FIL-16	0	0	0	0.84763822	78.2707297	0	0	0	0	18.29872	559400.909	4	2.5	279296.297	9130.90
FIL0	0	0	0	0.23723737	29.1778987	0	0	0	0	20.16381	208534.446	20	2.5	264771.19	9744.20
FIL+28	0	0	0	0.34319753	34.619613	0	0	0	0	29.88582	247426.378	48	2.5	295145.07	10388.79
FIL+45	0	0	0	0.4309035	43.785308	0	0	0	0	28.98737	312933.6	65	2.5	332326.90	11731.07
FIL+67	0	0	0	0.41020573	40.4377831	0	0	0	0	25.51641	289008.84	87	2.5	322118.66	9478.54
FIL+80	0	0	0	0.44141292	48.8256841	0	0	0	0	26.97709	348957.168	100	2.5	379286.60	12813.79
FIL+100	0	0	0	0.37678276	42.3885552	0	0	0	0	23.65552	302951.008	120	2.5	395999.49	14103.31
FIL+127	0	0	0	0.46314244	48.2277204	0	0	0	0	23.50414	344683.522	147	2.5	421998.72	14967.86
FIL+150	0	0	0	0.5533492	51.2681967	0	0	0	0	19.52322	366413.806	170	2.5	455407.25	13365.95
FIL+184	0	0	0	0.61687036	53.3276616	0	0	0	0	17.35842	381132.801	204	2.5	543085.68	18047.60
FIL+226	0	0	0	0.63486841	58.1517759	0	0	0	0	22.70682	415610.746	246	2.5	610722.83	21450.91
FIL+256	0	0	0	0.46694962	43.2967675	0	0	0	0	17.12141	309442.001	276	2.5	697262.78	23727.81
