# Supplementary material for: Temporal and spatial earthquake clustering revealed through comparison of millennial strain-rates from 36Cl cosmogenic exposure dating and decadal GPS strain-rate
Source: Sci Rep. 2021 Dec 2;11:23320. doi: 10.1038/s41598-021-02131-3 (PMC8639784; doi:10.1038/s41598-021-02131-3)
Supplement: Supplementary file 7 — Supplementary Information 7. [file 41598_2021_2131_MOESM7_ESM.pdf]

SUPPLEMENT S2b.iv

Input values used within the “mag_field file” in the Beck et al. (2018) Matlab code

Fault name	Spallation	Muons capture
Milesi	1.13	1.016
Malakasa	1.24	1.072
Fili	1.73	1.307
