# Supplementary figures and images for: Temporal and spatial earthquake clustering revealed through comparison of millennial strain-rates from 36Cl cosmogenic exposure dating and decadal GPS strain-rate
Source: Sci Rep. 2021 Dec 2;11:23320. doi: 10.1038/s41598-021-02131-3 (PMC8639784; doi:10.1038/s41598-021-02131-3)

# SUPPLEMENT S2c Full Bayesian results

## Milesi fault

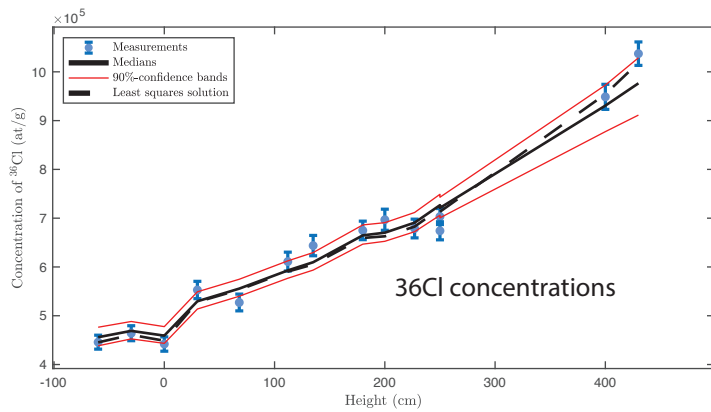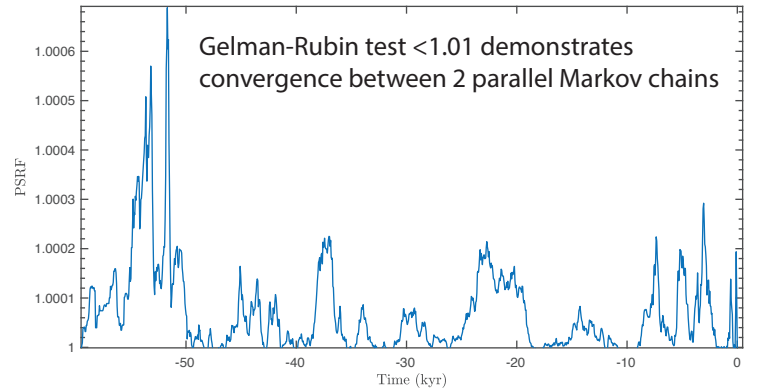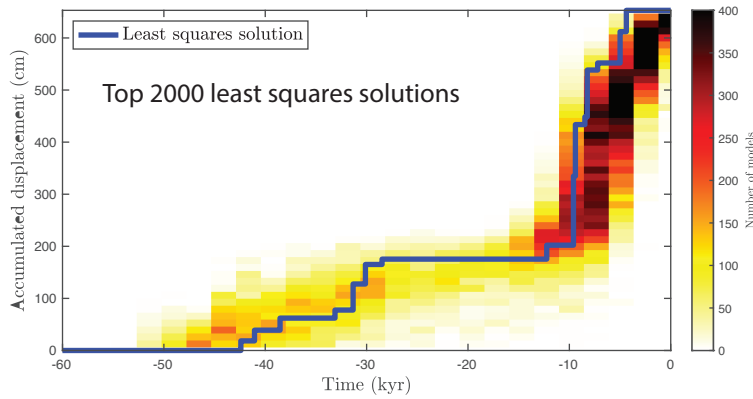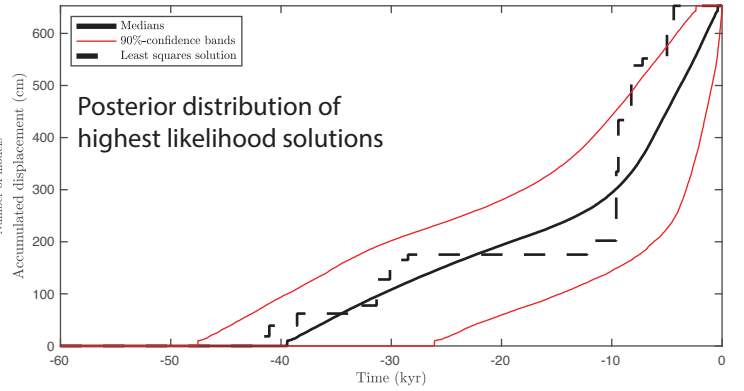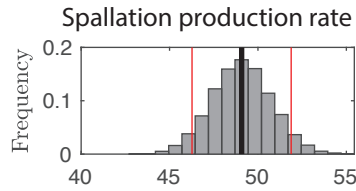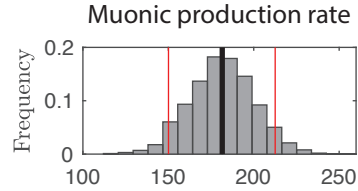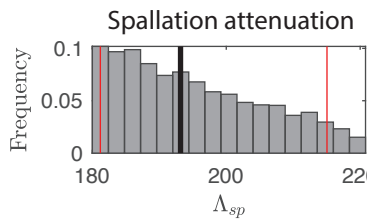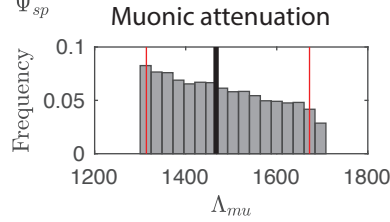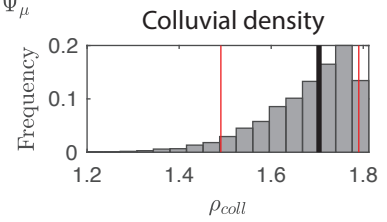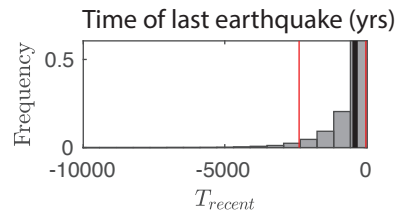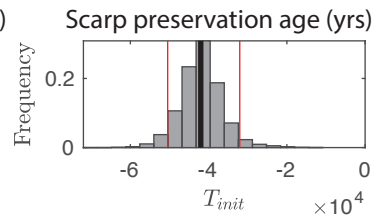

# Malakasa fault

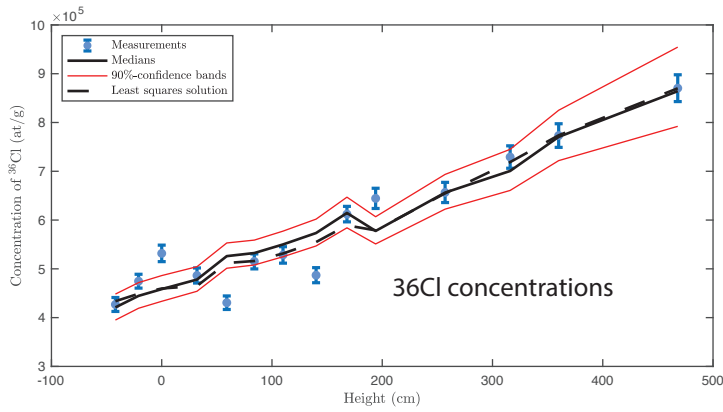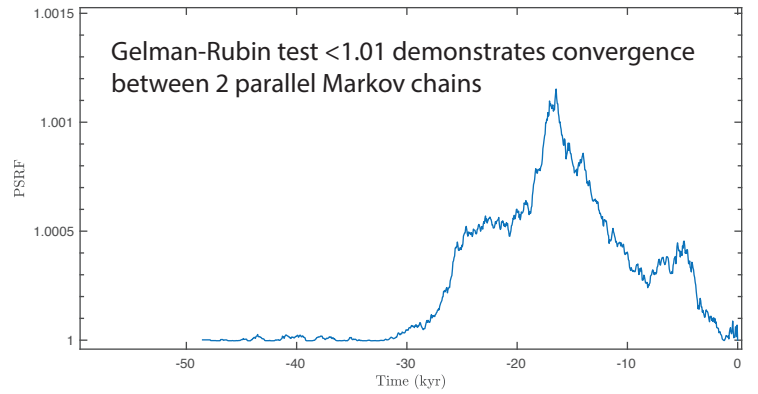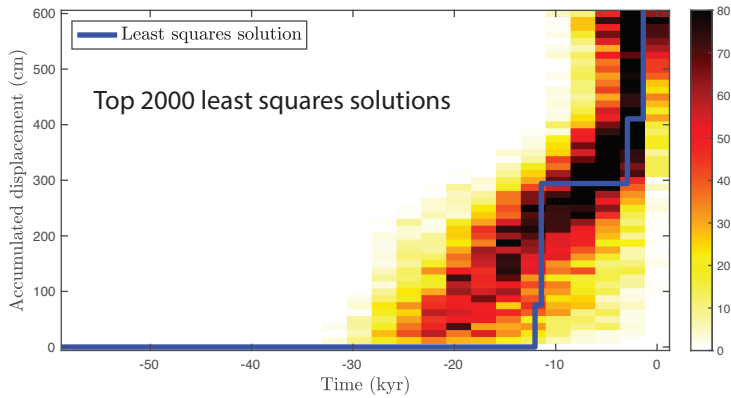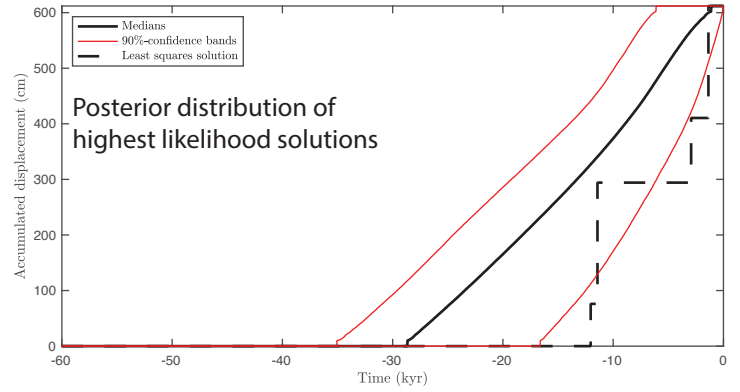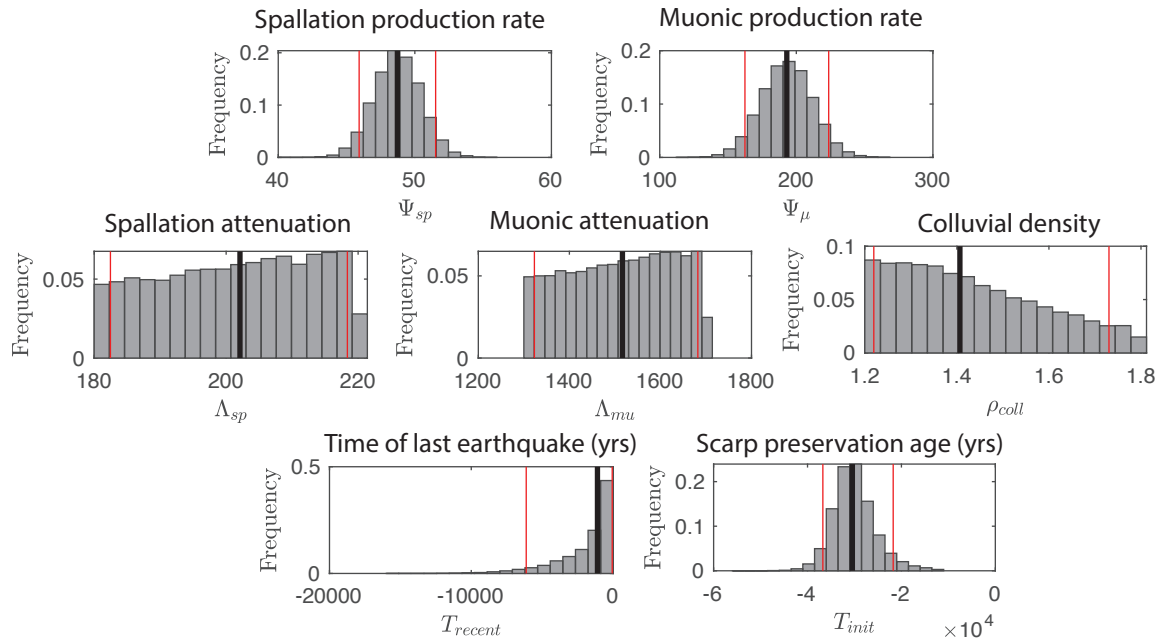

# Fili fault

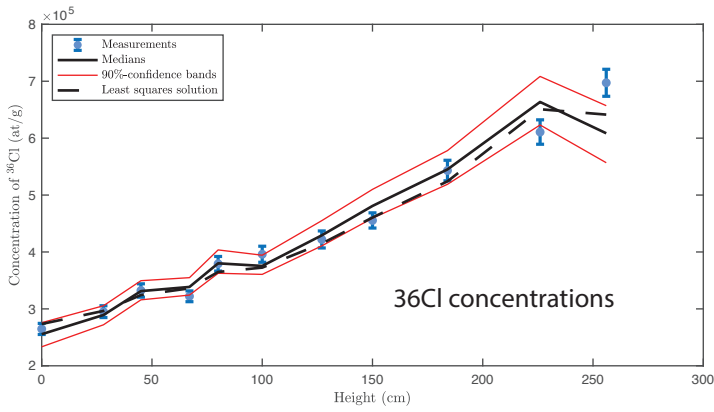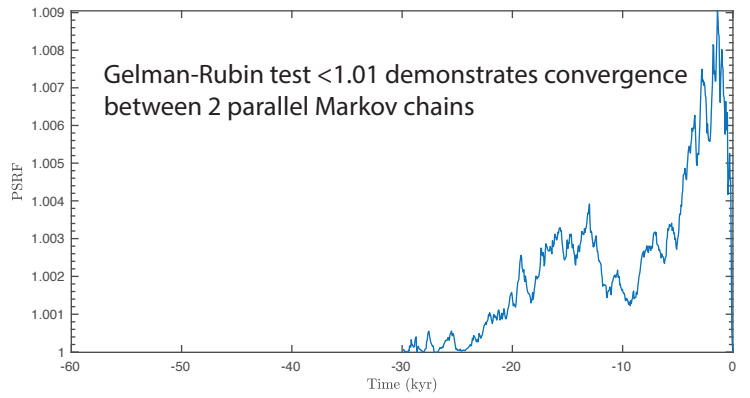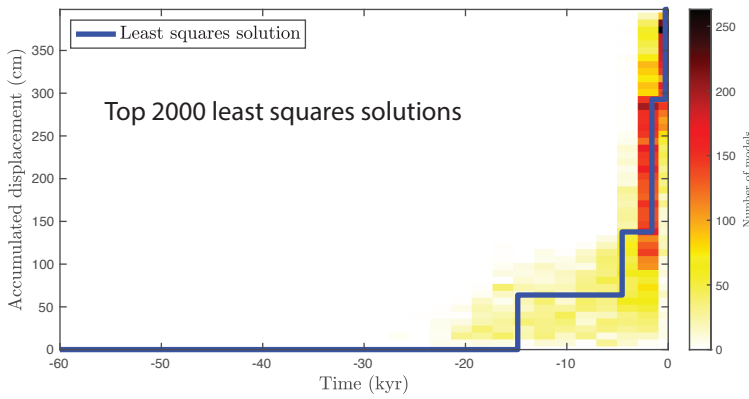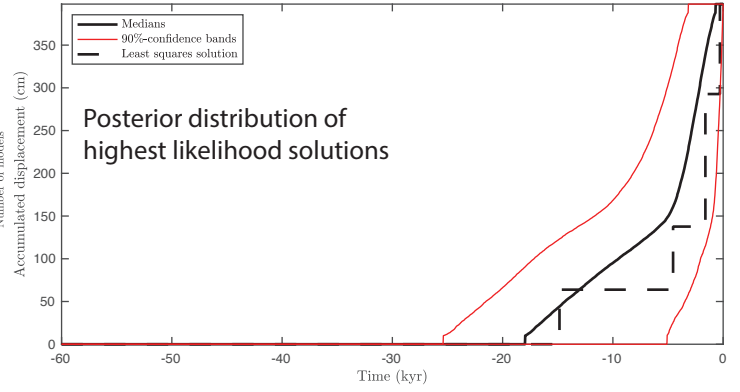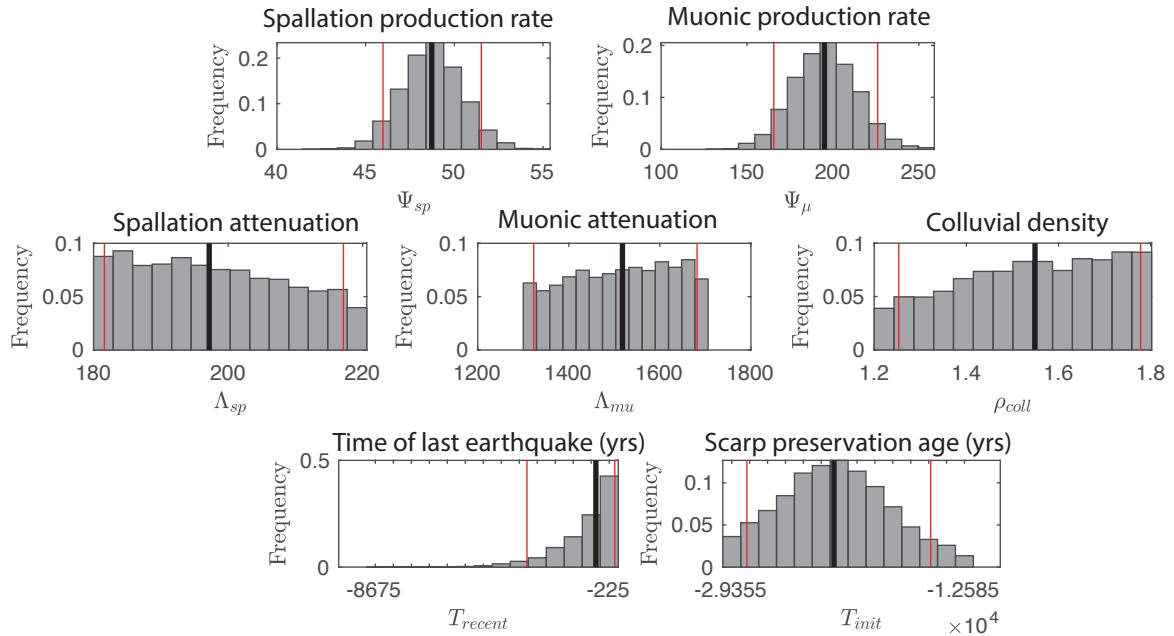

Supplement: Supplementary file 8 — Supplementary Information 8. [file 41598_2021_2131_MOESM8_ESM.pdf]
