# Supplementary material for: Temporal and spatial earthquake clustering revealed through comparison of millennial strain-rates from 36Cl cosmogenic exposure dating and decadal GPS strain-rate
Source: Sci Rep. 2021 Dec 2;11:23320. doi: 10.1038/s41598-021-02131-3 (PMC8639784; doi:10.1038/s41598-021-02131-3)
Supplement: Supplementary file 9 — Supplementary Information 9. [file 41598_2021_2131_MOESM9_ESM.pdf]

Malakasa fault

Supplement S2d

Geological cross-sections across the Malakasa and Fili faults.

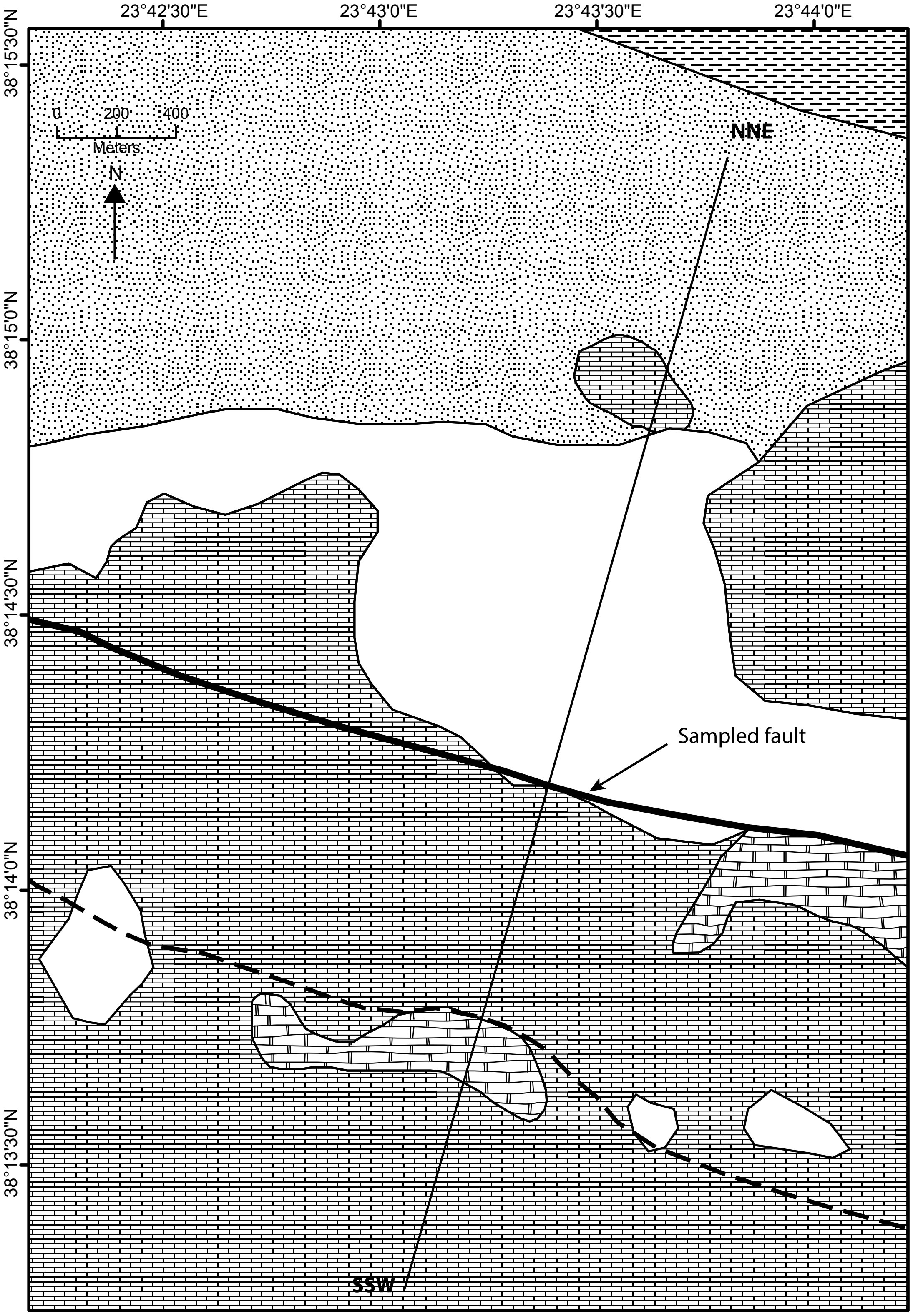

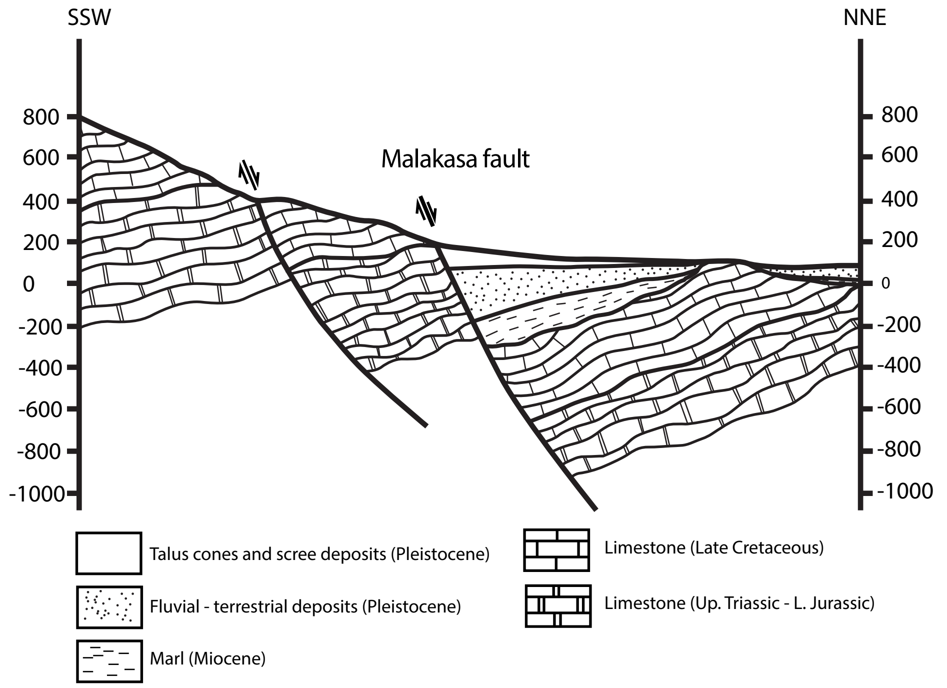

Geology is modified after Parginos, D., et al., 2007, 1:50000 Geological Map "Chalkida". IGME, Athens.

Fili fault

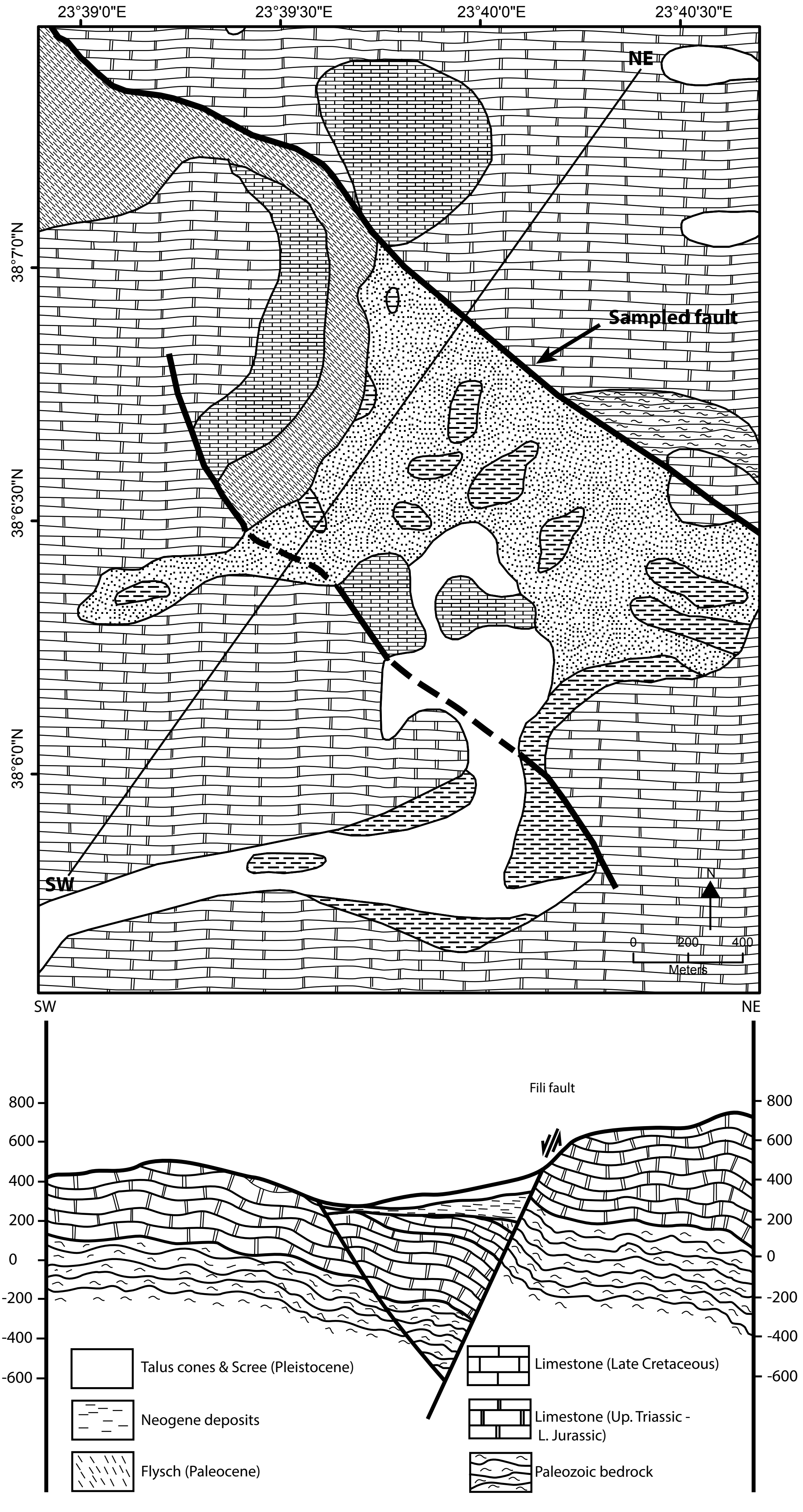

Geology is modified after Katsikatsos, G., et al., 1986, 1:50000 Geological Map "Athina-Elefsis". IGME, Athens
