# Supplementary material for: Temporal and spatial earthquake clustering revealed through comparison of millennial strain-rates from 36Cl cosmogenic exposure dating and decadal GPS strain-rate
Source: Sci Rep. 2021 Dec 2;11:23320. doi: 10.1038/s41598-021-02131-3 (PMC8639784; doi:10.1038/s41598-021-02131-3)

Supplement S3a

^{36}Cl data and implied slip versus time history for the Pisia fault in central Greece (data from Mechernich et al. 2018) and the Fiamignano fault in central Italy (data from Cowie et al. 2017 and Beck et al. 2018). The top 10,000 least squares solutions are shown in (ii) and (iv).

i) Fiamignano fault
36Cl concentration v height on the fault plane

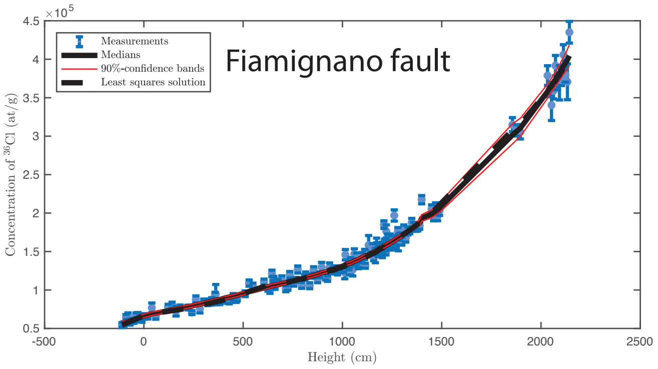

ii) Slip v time history for the Fiamignano fault inferred using the MCMC code BED v1 from Beck et al. (2018)

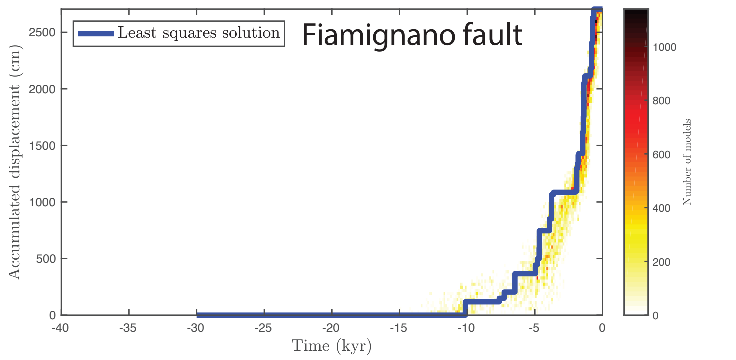

iii) and iv) Slip v time history for the Fiamignano fault since 4 ka and the timings of 2 earthquakes thought to have occurred on this fault from historical records. Note the ensemble of least squares solutions appear to identify periods of rapid slip coincident with these earthquakes. iii) is the posterior distribution; iv) is least squares. 847 AD and 1349 AD are two historical earthquakes that damaged Rome.

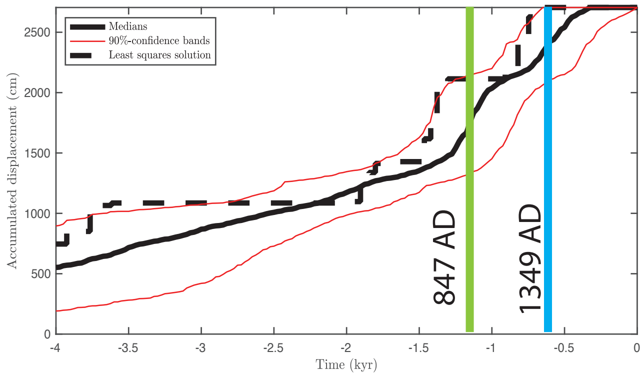

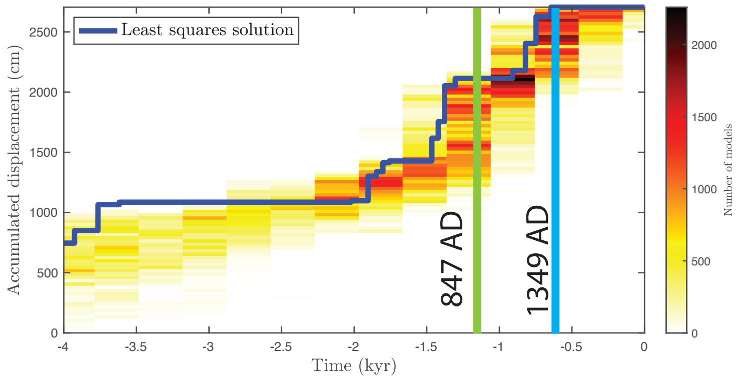

v) Pisia fault ^{36}Cl concentration v height on the fault plane. vi) Derived slip v time using the Beck et al. (2018) code.

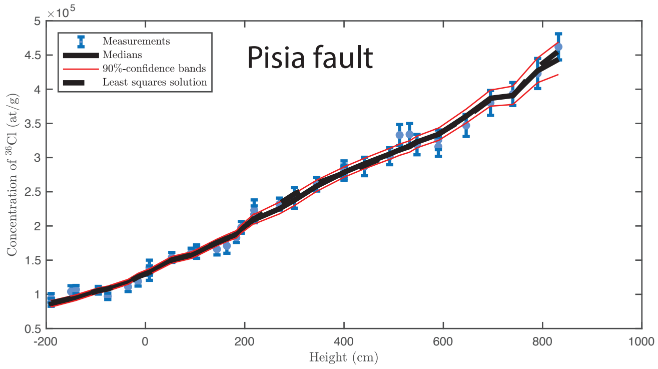

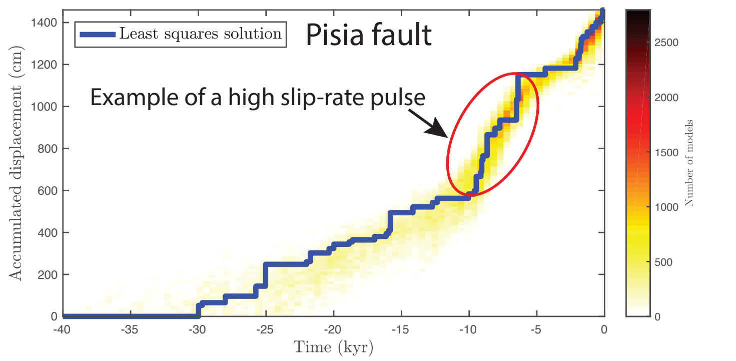

Supplement: Supplementary file 11 — Supplementary Information 11. [file 41598_2021_2131_MOESM11_ESM.pdf]
