# Supplementary material for: Temporal and spatial earthquake clustering revealed through comparison of millennial strain-rates from 36Cl cosmogenic exposure dating and decadal GPS strain-rate
Source: Sci Rep. 2021 Dec 2;11:23320. doi: 10.1038/s41598-021-02131-3 (PMC8639784; doi:10.1038/s41598-021-02131-3)
Supplement: Supplementary file 12 — Supplementary Information 12. [file 41598_2021_2131_MOESM12_ESM.pdf]

Supplement 3b Comparison of results for the Pisia Fault using three different codes. Relatively slow-slip separated by a period of more rapid slip at ~7-10 ka is resolved by all 3 codes.

Results from the Schlagenhauf et al. 2010 code

Results from the Cowie et al. 2017 code

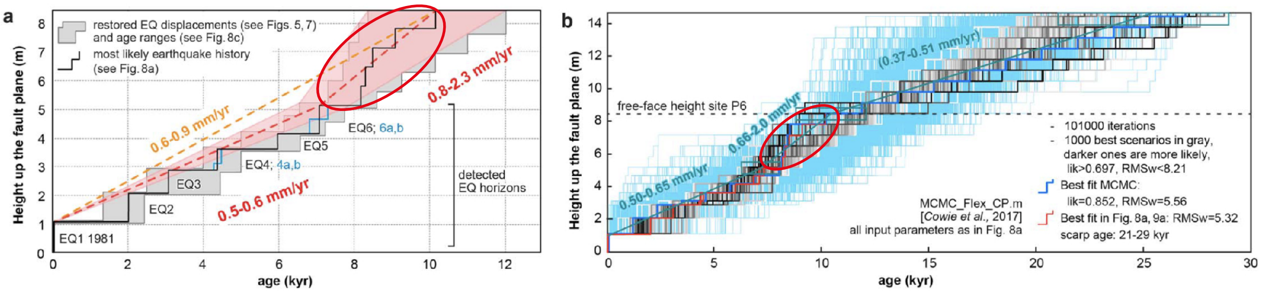

Figure 9. The exhumation history of the free-face at site P6. (a) Modeling results of the manual iteration (see Figures 8a and 8c). The slip rate was 0.5–0.6 mm/yr for the last ~7.3 kyr (1.1–5.15 m). For the upper part of the free-face (5.15–8.45 m; hypothetical earthquake offsets) the exhumation occurred at a significantly higher rate. (b) Earthquake history modeling scenarios using the MCMC Matlab® code of Cowie et al. (2017). The range of slip rates for the 100 most likely models are given in turquoise. Please note that the apparent slip history of the degraded scarp and the scarp age are hypothetical due to significant erosion, sedimentation at the scarp base, and a lack of cosmogenic data.

Results from the Beck et al. 2018 code

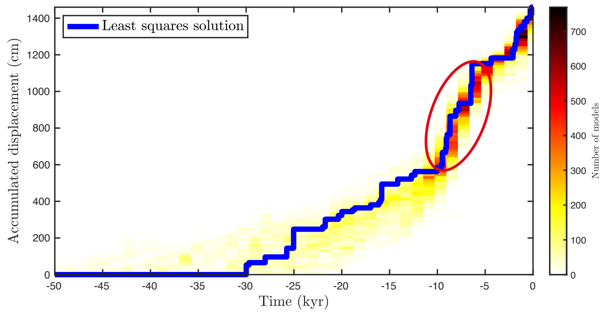

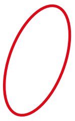

= Earthquake cluster resolved by all 3 codes at ~10-7 ka
