# Supplementary material for: Temporal and spatial earthquake clustering revealed through comparison of millennial strain-rates from 36Cl cosmogenic exposure dating and decadal GPS strain-rate
Source: Sci Rep. 2021 Dec 2;11:23320. doi: 10.1038/s41598-021-02131-3 (PMC8639784; doi:10.1038/s41598-021-02131-3)

Supplement S3c: Comparison of results from four runs of the Beck et al. (2018) to examine whether recognition of clustering can be considered robust given discontinuous sampling and varying the history of initial ^{36}Cl production. The earthquake cluster at ~10-7 ka is resolved in all 4 runs suggesting the choice of discontinuous sampling and initial ^{36}Cl production is robust. Panels from top to bottom are ensembles of the top 2000 least squares solutions after 50% burn-in with the least squares shown, solely the least squares ensembles, ^{36}Cl concentrations, and highest likelihood solutions after 50% burn-in.

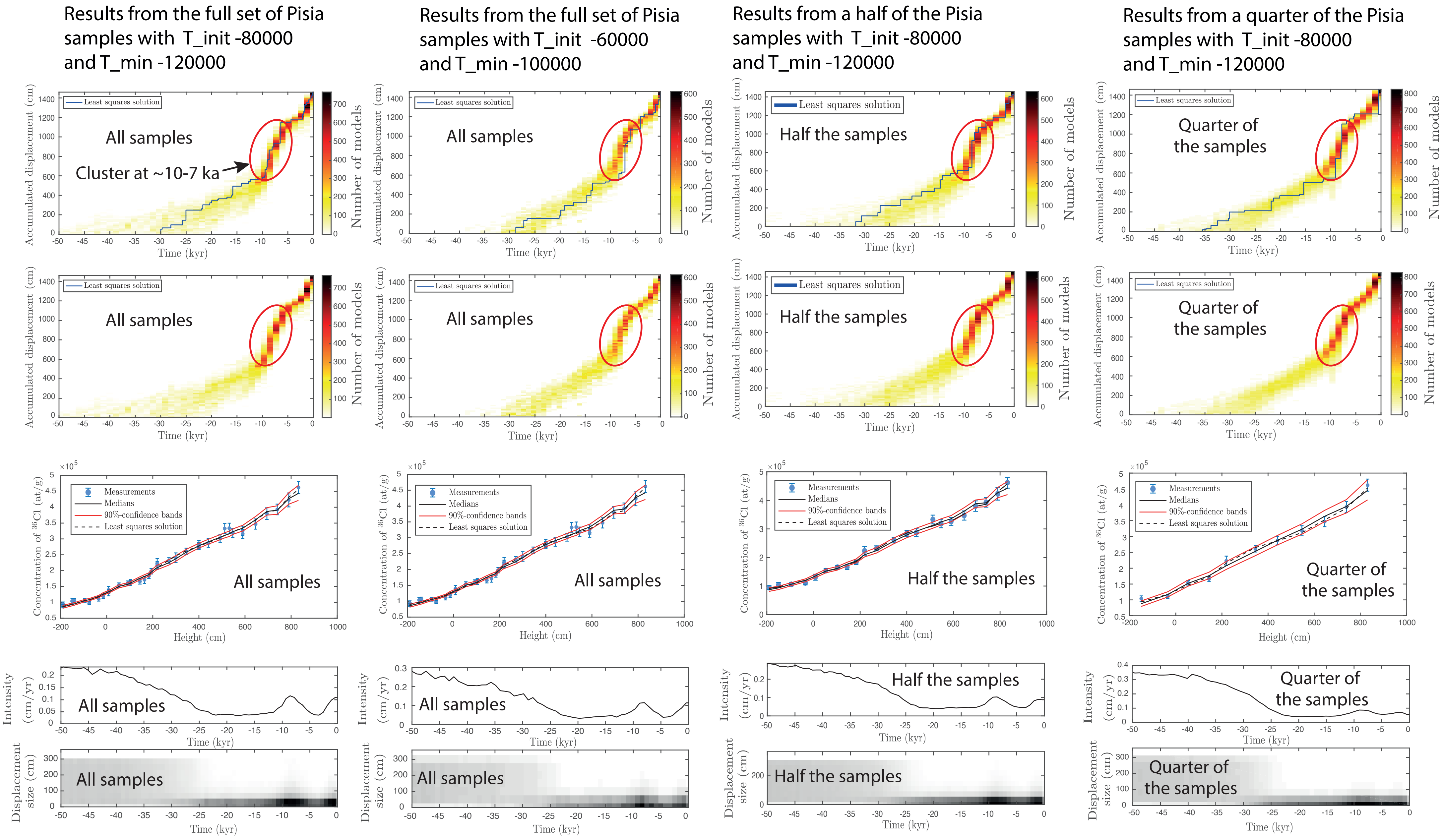

Supplement: Supplementary file 13 — Supplementary Information 13. [file 41598_2021_2131_MOESM13_ESM.pdf]
