# Supplementary material for: Temporal and spatial earthquake clustering revealed through comparison of millennial strain-rates from 36Cl cosmogenic exposure dating and decadal GPS strain-rate
Source: Sci Rep. 2021 Dec 2;11:23320. doi: 10.1038/s41598-021-02131-3 (PMC8639784; doi:10.1038/s41598-021-02131-3)

Supplement S6: Plots showing that the range of iteration of the production rates and colluvial densities for the actual run is larger than the variation in these parameters implied by difference in the colluvial composition

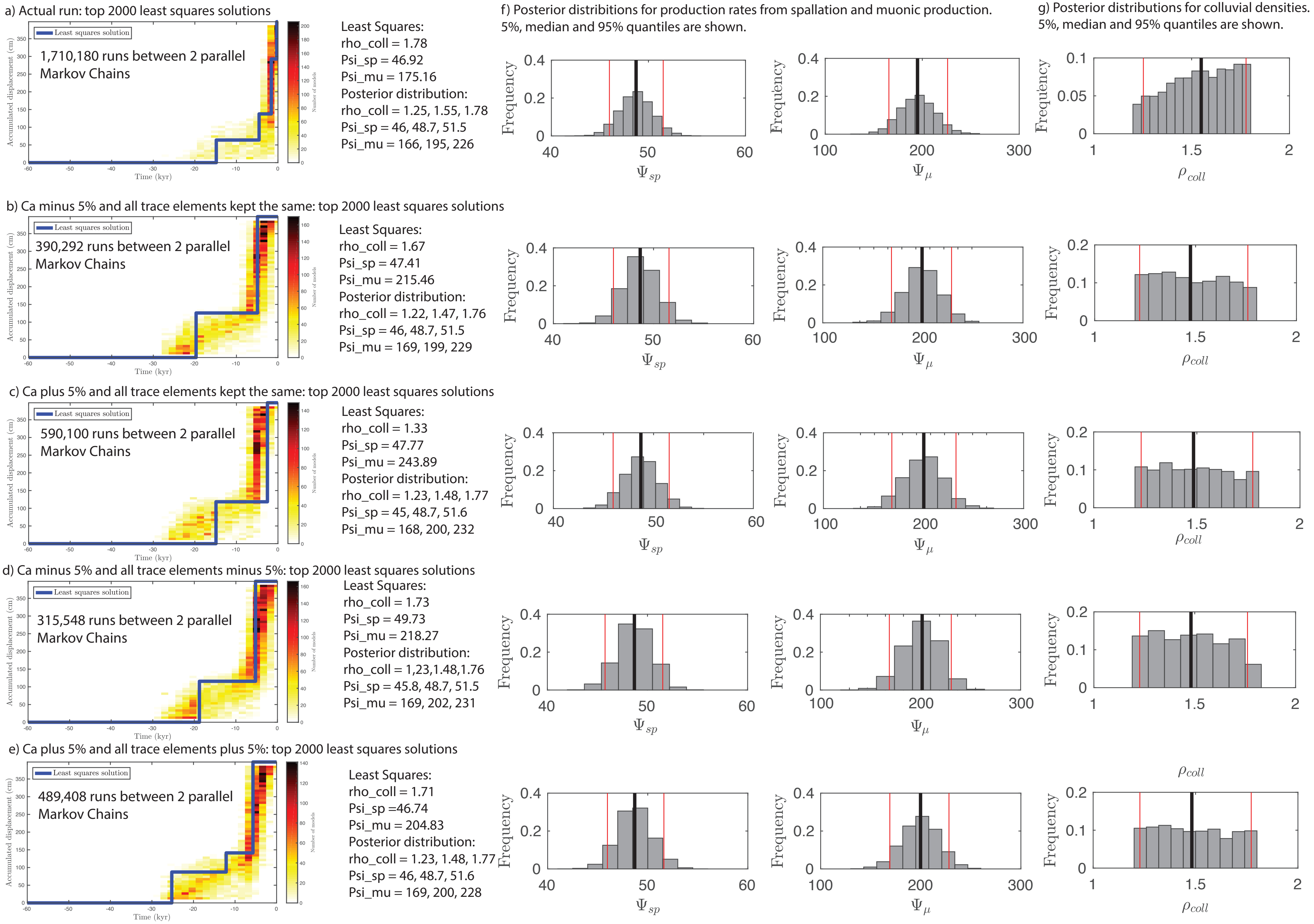

Supplement: Supplementary file 16 — Supplementary Information 16. [file 41598_2021_2131_MOESM16_ESM.pdf]
